# Clustering of Cerebral Microbleeds in Cerebral Amyloid Angiopathy: A Flow Territory‐Based Analysis

**DOI:** 10.1002/jmri.70454

**Published:** 2026-07-25

**Authors:** Manon R. Schipper, Reinier G. J. van der Zwet, Jelle J. Goeman, Thijs W. van Harten, Emma A. Koemans, Marieke J. H. Wermer, Marianne A. A. van Walderveen, Matthias J. P. van Osch

**Affiliations:** ^1^ Department of Radiology Leiden University Medical Center Leiden the Netherlands; ^2^ Department of Neurology Leiden University Medical Center Leiden the Netherlands; ^3^ Department of Biomedical Data Sciences Leiden University Medical Center Leiden the Netherlands; ^4^ Department of Neurology Massachusetts General Hospital, Harvard Medical School Boston Massachusetts USA; ^5^ Department of Neurology University Medical Center Groningen Groningen the Netherlands

**Keywords:** amyloid‐β, cerebral amyloid angiopathy, cerebral flow territories, cerebral hemorrhages, cerebral microbleeds, small vessel disease

## Abstract

**Background:**

Cerebral microbleeds (CMBs) are a key imaging marker of cerebral amyloid angiopathy (CAA), but their individual spatial distribution and potential clustering remain poorly understood.

**Purpose:**

This study assessed whether CMBs exhibit patient‐specific, flow territory‐based clustering in sporadic (sCAA) and Dutch‐type hereditary CAA (D‐CAA). The arterial flow territory subdivision was chosen, considering the predilection of CMB location in the posterior cortex—not limited to the occipital lobe—and the prominent vascular role in CAA.

**Study Type:**

Retrospective cross‐sectional study.

**Population:**

Thirty‐three participants (17 (52%) female, 65 ± 12 years old, 19 sCAA; 14 D‐CAA) without symptomatic intracerebral hemorrhage (sICH) and ≥ 1 CMB were included for the main analysis.

**Field Strength/Sequence:**

3 T MRI susceptibility‐weighted imaging, multi‐echo 3D gradient echo.

**Assessment:**

Spatial CMB patterns were evaluated by comparing observed versus expected CMB density within covariate‐matched subgroups, accounting for age, sex, CAA‐type, smoking, and hypertension. CMBs were manually annotated and assigned to six arterial flow territories (left/right ACA, MCA, PCA) using a standardized atlas. A sensitivity analysis was performed on all participants (including sICH+ participants).

**Statistical Tests:**

Expected values were calculated by multiplying flow territory CMB proportions per covariate‐matched subgroup. Unweighted individual chi‐square statistics were calculated, and statistical significance was assessed via permutation testing (10,000 replications) per covariate group.

**Results:**

Across covariate‐matched subgroups, observed CMB counts significantly deviated from expected distributions, indicating substantial interindividual variability and within‐participant clustering (*χ* ^2^ = 6920). The effect remained significant when including sICH+ participants (*χ* ^2^ = 102,027, *n* = 85; 44 sCAA; 41 D‐CAA). CMB densities differed across flow territories, with highest median densities in the right PCA, right MCA, and left PCA (0.173 (IQR = 0.387), 0.121 (IQR = 0.238), and 0.113 (IQR = 0.389) counts per cm^3^, respectively).

**Data Conclusion:**

These findings indicate CMBs in CAA are not identically distributed but show patient‐specific clustering within flow territories, suggesting variable regional vulnerability, potentially influenced by local pathology or vascular anatomy.

**Evidence Level:**

1.

**Technical Efficacy:**

Stage 2.

## Plain Language Summary

Cerebral amyloid angiopathy is a cerebral small vessel disease that is characterized by vascular accumulation of the protein amyloid‐beta. This accumulation causes vessel rigidness and brain damage, eventually in the form of hemorrhages. This study investigates whether one type of those hemorrhages, cerebral microbleeds, tends to cluster together and whether this clustering is patient‐specific. The assessment of clustering has been based on brain regions that are perfused by the different major cerebral arteries. The results show that cerebral microbleeds in cerebral amyloid angiopathy tend to cluster together, with patient‐specific clustering patterns.

## 1 Introduction

Cerebral amyloid angiopathy (CAA) is a neurological disease caused by amyloid‐beta (Aβ) accumulation in cerebral cortical and leptomeningeal blood vessels [1]. Clinically, non‐hereditary sporadic CAA (sCAA) is characterized by cognitive decline, transient focal neurological episodes, and, most notably, intracerebral hemorrhages (ICHs) [2]. Dutch‐type hereditary CAA (D‐CAA) is the most prevalent autosomal dominant form of CAA, caused by a point mutation in the APP gene (APP 693) [3]. It has an analogous presentation to sCAA, albeit with an earlier onset and more aggressive disease course [3, 4].

One of the hallmark biomarkers of CAA is the presence of lobar cerebral microbleeds (CMB), on susceptibility‐weighted imaging (SWI), which are thought to be small deposits of hemosiderin [1]. Lobar CMBs are considered a marker of advanced CAA, according to the previously proposed disease stages of CAA, indicating structural cortical vessel fragility [5]. Nearly all patients with sCAA or D‐CAA who show hemorrhagic lesions have progression of CMBs within 1 year, reflecting ongoing vascular damage and disease progression [6].

CAA pathology is not uniformly distributed across the brain. Studies have shown that CMBs in CAA are almost exclusively localized in cortical gray matter, with a predilection for posterior brain regions, especially, but not limited to, the occipital lobe [7, 8, 9, 10]. Similarly to CMBs, ICHs also occur most frequently in the occipital lobe, even when adjusted for lobar volume [11]. These findings align with evidence from pathological and positron emission tomography (PET) studies, which indicate that Aβ deposition is most pronounced in the occipital lobe [12, 13, 14].

While the spatial distribution of CMBs in CAA has been extensively studied at the group level, clustering and individual variability remain largely unexplored. In one study, individual clustering of new symptomatic and asymptomatic hemorrhages within lobes has been observed [7]. Anecdotally, in our study cohort we observed clear asymmetries, for example, between the right and left hemispheres, in CMB distribution in our participants while scanning participants. These asymmetries might reflect disease processes affecting different parts of the arterial tree to varying degrees. In these cases, parts of the arterial tree may have increased vulnerability, enhancing the effects from vascular Aβ or accelerating its deposition. While Aβ deposition affects the arterial vasculature, patterns based on flow territories have, to our knowledge, not yet been studied in the context of CAA. In addition, predilection of other CAA markers does not appear to be restricted to specific cerebral lobes, indicating that other subdivisions may play a role. Contrasting, asymmetries may also arise from factors independent of vascular anatomy, such as local inflammation, which is associated with CMB clusters [15].

This study aimed to determine whether CMBs tend to cluster together within flow territories. It also aimed to inspect CMB densities across the different flow territories.

## 2 Methods

### 2.1 Patient Selection

Participants with sCAA and D‐CAA were included in this cross‐sectional study. Participants were retrieved from the prospective natural history study on sporadic CAA (FOCAS) and D‐CAA (AURORA); all patients from one timepoint with available data were included. Inclusion criteria for FOCAS were age ≥ 55 years and a CAA diagnosis based on the modified Boston criteria based on 1.5 or 3.0 Tesla (3 T) MRI. Inclusion criteria for AURORA were age ≥ 18 years, no contraindications for 3 T MRI, and a DNA‐proven APP mutation. The additional inclusion criterion for the current study was the presence of at least one CMB on SWI. Exclusion criteria for the current study were history of CAA related inflammation (given its known clustering effect [15]) and missing covariate data. The primary focus of the study was on participants without a history of symptomatic ICH (sICH) to minimize the potential influence of sICH‐related local effects on CMB distribution. Information on medical history and vascular risk factors were retrieved from medical records and medical questionnaires administered during study visits. The FOCAS and AURORA studies were performed following the Helsinki protocol and approved by the Medical Ethics Committee Leiden the Hague Delft. Written informed consent was obtained from all participants before enrollment.

### 2.2 Image Acquisition

MRI scans in the FOCAS and AURORA studies were acquired on a whole‐body 3 T magnetic resonance system (Philips, Best, the Netherlands) with a standard 32‐channel head coil. The scan protocol has been described previously [6]. For the current study, SWI was acquired with the following parameters: repetition time = 31 ms, number of echos = 4, first echo time/echo spacing = 7.2/6.2 ms, flip angle 17°, 140 slices with no interslice gap and field of view = 230 × 190 × 140 mm, with acquisition voxel size = 0.6 × 0.6 × 2.0 mm, resulting in a scan duration of ~6 min.

### 2.3 Microbleed Localization Pipeline

CMBs were scored on SWI if smaller than 10 mm in diameter, also including non‐homogeneous lesions, following the STRIVE criteria (2.0) [16], by one observer with 7 years of experience in neuroimaging research and cerebral microbleed assessment (EAK). A neuroradiologist with over 20 years of experience (MAAvW) supervised the training of the observer and provided expert consultation in cases of uncertainty. CMBs were manually annotated for each participant, and CMB world coordinates were stored in a CMB map [17, 18]. A cerebral flow territory atlas (excluding the cerebellum) subdivided the left and right hemispheres, and the arteries into an anterior, middle, and posterior part (ACA, MCA, and PCA, respectively) [19]. Per participant, each flow territory region was binarized and registered to the SWI using FSL's linear (FLIRT) and non‐linear registration (FNIRT) [20, 21, 22]. To minimize classification errors that may result from the registration and preserve size of the original atlas, the registered regions were thresholded at 0.5 in accordance with FSL's common practices.

The CMB map was overlayed onto the registered and thresholded flow territories to extract the corresponding locations per CMB. If, due to thresholding, no immediate overlap was present for a CMB, the Euclidean distance between the CMB and each region was determined. The flow territory with the minimal Euclidean distance was then assumed to be the corresponding flow territory for this CMB [18]. The cerebral flow territory atlas excluded the cerebellum, as a different flow territory feeds the cerebellum. Therefore, cerebellar CMBs were not included for the current analyses. Finally, regional volumes were derived from registered flow territory atlases. CMB densities were calculated by dividing the CMB count per flow territory by the volume of the corresponding flow territory.

### 2.4 Data Analysis

For the primary analysis participants without sICH were included. Individual patient covariate profiles were characterized based on mutation status, hypertension, smoking, sex, and age (in bins of 10 years). Participants were considered to have the same covariate profile if they were identical on all considered covariates. To test whether the clustering pattern of CMBs was consistent across participants, expected counts were calculated under the null hypothesis of no patient‐specific variation within the covariate profile groups. The frequency of CMBs in each flow territory was determined separately for each unique combination of covariates (i.e., using a stratified permutation test) [23]. For every individual patient, the expected number of CMBs for each of the six flow territories (left and right ACA, MCA, and PCA) was estimated by multiplying the participants' total CMB count by the averaged fraction of CMBs observed in that flow territory stratified within the specific covariate group. This resulted in an expected spatial distribution based solely on CMB count and covariate‐group averages, under the assumption that no patient‐specific spatial variation exists.

For the primary outcome, an unweighted chi‐square statistic was calculated by summing the squared differences between the observed and expected counts across participants and territories. An unweighted chi‐square statistic was calculated, as this approach can better handle large, sparse contingency tables, as the usual chi‐square test loses power quickly when some expected counts are small, as expected here [24]. To evaluate the level of statistical significance, a permutation test was applied by generating a null distribution of unweighted chi‐square statistics under the assumption that any spatial clustering of CMBs is unrelated to patient identity. This was achieved by randomly reassigning patient labels to individual CMBs within each covariate combination, thus maintaining the total CMB count per patient and covariate group, but randomizing spatial assignments across individuals. This process was repeated 9999 times, with the unweighted chi‐square statistic recalculated for each permuted dataset to approximate the distribution of values expected under the null hypothesis of no patient‐specific pattern (see Supporting Information for a histogram). The primary outcome *p* value was obtained by comparing the actual observed unweighted chi‐square statistic—that was obtained without reassignment of patient labels to the individual CMBs—to this null distribution and calculated as one plus the number of permuted unweighted chi‐square statistics that exceed the actual value, divided by 9999 + 1 [25]. A visual representation of the statistical analysis for the primary outcome is presented in Figure 1.

**FIGURE 1:**
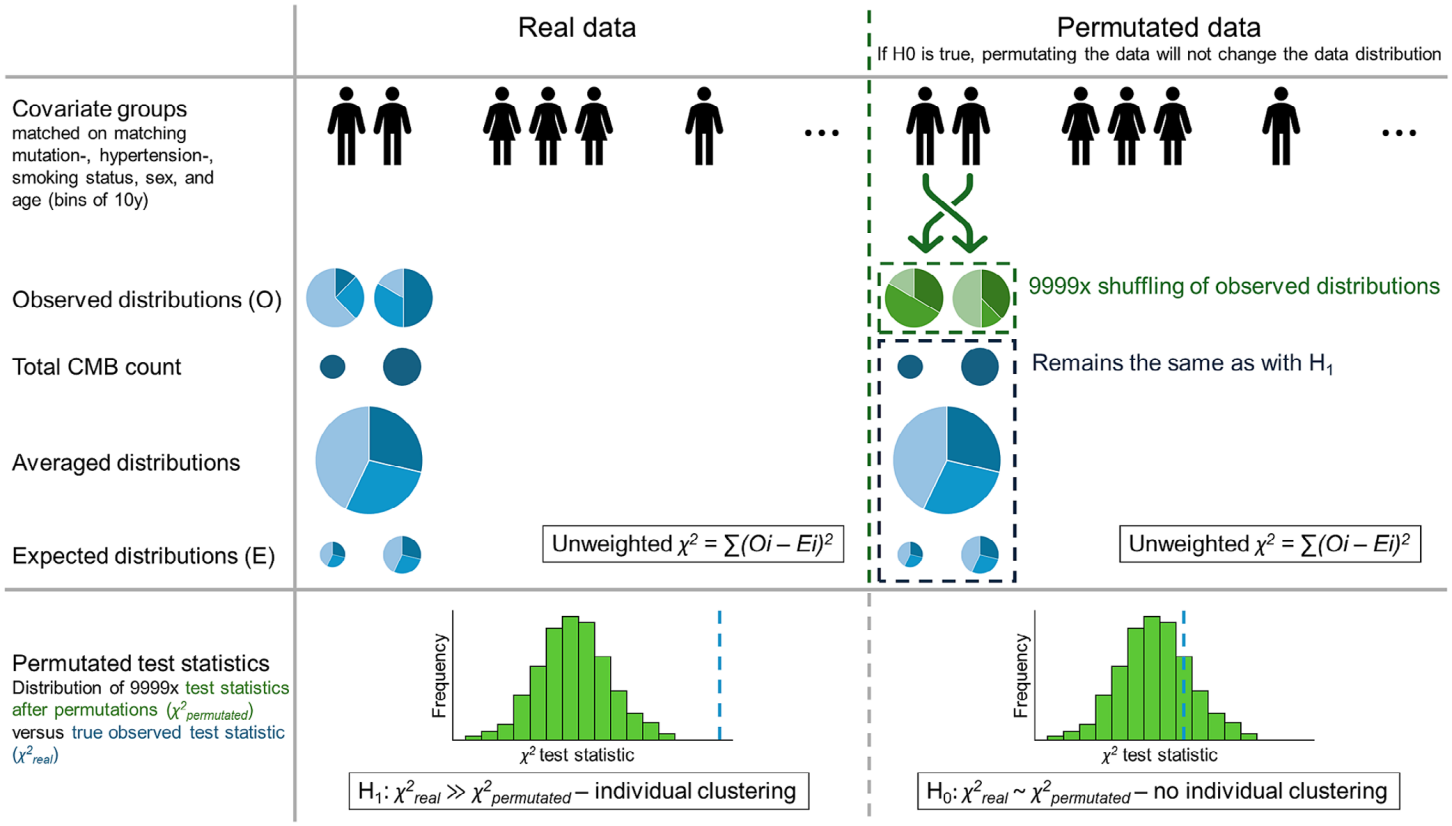
Visual representation of the unweighted Chi‐square tests to assess clustering within flow territories and between individuals. The left side shows the unweighted Chi‐square test on the real data—without shuffling of individual cerebral microbleeds (CMBs)—to test whether the observed observations are different from the expected distributions. The right side shows the unweighted Chi‐square tests on the permutated data—after shuffling the individual CMBs 9999 times within the covariate‐group—to create a distribution of Chi‐square statistics to test H_1_ of individual clustering.

To assess the robustness of the findings, a sensitivity analysis that included the full sample was conducted, that is, incorporating participants with sICH. Also, a subset of the data (84% of the total sample and 88% of the participants without sICH) was scored by a second observer to investigate CMB rating agreement. Inter‐rater reliability between the two raters was calculated using a two‐way random‐effects, absolute‐agreement, single‐measures model. Finally, we assessed sensitivity to outliers by checking whether results remained significant if any one or two covariate groups were removed.

Additionally, differences in CMB density between the flow territories were assessed using the non‐parametric repeated measures Friedman test. If the Friedman test showed a significant difference in the CMB density between the flow territories, the pair‐wise Wilcoxon signed‐rank test was performed, with Holm's method of correcting for multiple testing.

All statistical analyses were conducted in R (version 4.4.0) with *α* set at 0.05. Only participants with complete data were included in the analysis; no imputation was performed for missing values (complete case analysis).

The Strengthening the Reporting of Observational Studies in Epidemiology (STROBE) reporting guideline was followed. Further details on initial data analysis can be found in the Supporting Information.

## 3 Results

### 3.1 Participant Characteristics

The total sample consisted of 85 participants (36 (42%) female, 65 ± 11 years old, 44 sCAA, 41 D‐CAA; see Figure S1). The total number of CMBs in the total sample is 7033 for participants with sCAA and 10,726 for participants with D‐CAA. Additional patient characteristics for the total sample can be found in Table S1.

The main analysis was run on a subset of participants without history of sICH, resulting in a sample of 33 participants (17 (52%) female, 65 ± 12 years old, 19 sCAA, 14 D‐CAA). For the subset, the median number of CMBs per participant was 103 (IQR: 34–262) for sCAA and 64 (IQR: 5–156) for D‐CAA. Total number of CMBs was 3927 for participants with sCAA and 1750 for participants with D‐CAA. The mean age was 73.1 years (SD = 6.4) in the sCAA group and 54.1 years (SD = 9.0) in the D‐CAA group. Hypertension was present in 63% of sCAA versus 36% of D‐CAA participants. Other vascular risk factors, including hypercholesterolemia (58% vs. 29%) and history of smoking (100% vs. 71%), were also more prevalent in the sCAA group. The distribution of sex and type 2 diabetes mellitus was comparable between groups. Detailed descriptives for the participants without history of sICH can be found in Table 1.

**TABLE 1:** Participants characteristics of participants without sICH.

Characteristics	All participants (*n* = 33)	D‐CAA (*n* = 14)	sCAA (*n* = 19)
Age in years, mean (SD)	65.1 (12.1)	54.1 (9.0)	73.1 (6.4)
Female sex	17 (52%)	9 (64%)	8 (42%)
Hypertension	17 (52%)	5 (36%)	12 (63%)
Hypercholesterolemia	15 (45%)	4 (29%)	11 (58%)
Diabetes mellitus type 2	2 (6%)	1 (7%)	1 (5%)
Smoking, ever	29 (88%)	10 (71%)	19 (100%)
History of sICH	0 (0%)	0 (0%)	0 (0%)
Total CMB, median (IQR)	96 (23–234)	64 (5–156)	103 (34–262)
Cerebellar CMB, median (IQR)	2 (1–4)	2 (0–4)	2 (0–5)

Abbreviations: CMB, cerebral microbleeds; D‐CAA, Dutch‐type cerebral amyloid angiopathy; sCAA, sporadic cerebral amyloid angiopathy; sICH, symptomatic intracerebral hemorrhage.

The secondary and sensitivity analyses were run on the total sample, including participants with a history of sICH.

### 3.2 Spatial Distribution of CMB Densities Across Flow Territories

Across all participants, CMBs densities were significantly different throughout the territories (*χ* ^2^ = 43.481, df = 5). Results of the pair‐wise comparisons are shown in Table 2. Highest median densities were found in the right PCA, right MCA, and left PCA (0.173 (IQR = 0.057–0.445), 0.121 (IQR = 0.032–0.270), and 0.113 (IQR = 0.029–0.418), respectively; Figure 2). CMB density was significantly higher in the left PCA compared to the left ACA and left MCA, in the right MCA compared to the left ACA, and in the right PCA compared to the left ACA, left MCA, right ACA, and right MCA. No lateralization pattern was evident across the cohort when comparing left versus right matched flow territories (*p* = 0.665, for left ACA vs. right ACA, *p* = 0.127 for left MCA vs. right MCA, and p = 0.665 for left PCA vs. right PCA).

**TABLE 2:** *p* values from the Friedman test for group differences in CMB density.

	Left ACA	Left MCA	Left PCA	Right ACA	Right MCA
Left ACA (median = 0.078)	—	—	—	—	—
Left MCA (median = 0.092)	0.665	—	—	—	—
Left PCA (median = 0.113)	0.017	0.013	—	—	—
Right ACA (median = 0.100)	0.665	0.665	0.065	—	—
Right MCA (median = 0.121)	0.029	0.127	0.665	0.127	—
Right PCA (median = 0.173)	< 0.001	< 0.001	0.665	< 0.001	0.005

*Note:* CMB densities are in counts per cm^3^. *p* values are adjusted for multiple testing with Holm's method.

Abbreviations: ACA, anterior cerebral artery; CMB, cerebral microbleeds; MCA, middle cerebral artery; PCA, posterior cerebral artery.

**FIGURE 2:**
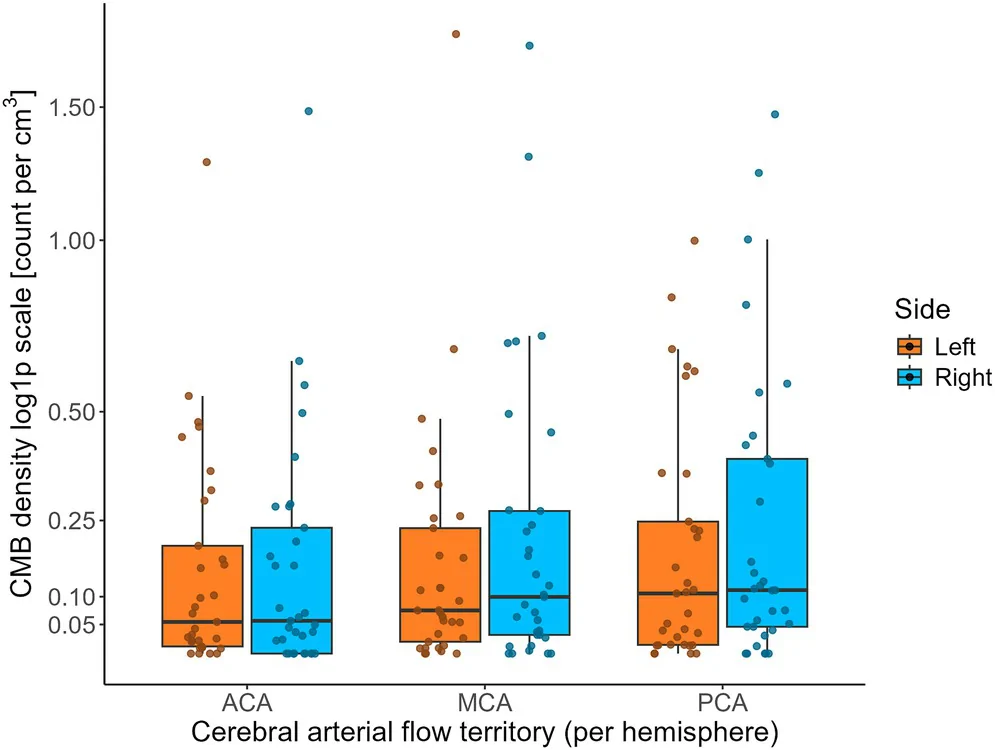
Boxplots of the (log1p) CMB density per flow territory, subdivided into left and right hemisphere. ACA, anterior cerebral artery; CMB, cerebral microbleeds; MCA, middle cerebral artery; PCA, posterior cerebral artery.

### 3.3 Interindividual Variation in Flow Territory Localization

Observed and expected CMB counts were compared per flow territory within covariate‐matched subgroups for participants with no history of sICH (*n* = 33, Table 1). The unweighted chi‐square statistic revealed a significant deviation from the null hypothesis of uniform distribution (*χ* ^2^ = 6920), indicating substantial interindividual variability within the covariate‐matched subgroups in spatial CMB patterns across the different flow territories (Figure S2A) and therefore an indication of clustering with interindividual variation in the region in which the clustering occurs. Covariate subgroup sizes are provided in Table S2.

Permutation testing confirmed the significance of this variability, with the observed unweighted chi‐square statistic far exceeding the 95th percentile of the null distribution. The mean *χ* ^2^ of the permutated null distributions was 979 (SD = 330).

Examples of six CMB patterns are shown in Figure 3.

**FIGURE 3:**
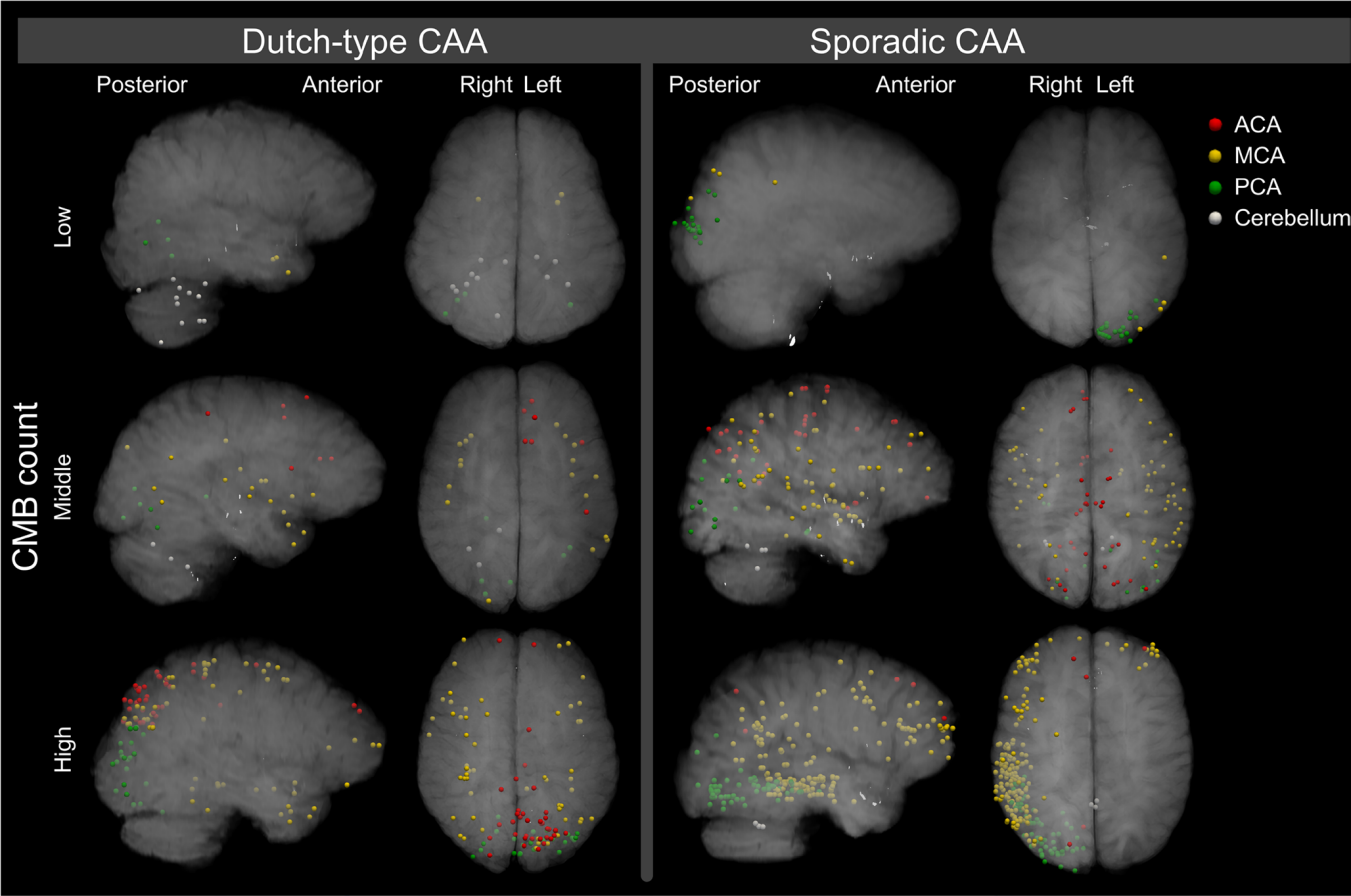
CMB projections onto rendered SWI images for a range of CMB counts, varying from low to high (~Q1 to Q3) counts from top to bottom for three participants with Dutch‐type CAA on the left (total CMB counts from top to bottom: 7, 31, and 114) and for three participants with sporadic CAA on the right (total CMB counts from top to bottom: 23, 115, and 234). Opacity of the markers indicates depth of the CMBs seen from right sagittal and top transverse orientation respectively. Cerebellar CMBs are not taken into account for the total count of CMBs, as they are not included in the analyses, however they are displayed for visualization purposes. The examples represent different clustering patterns in individual participants. From these examples, the most pronounced clustering is seen in the top right (with a main cluster in the left PCA) and in the bottom right representation (with a strong lateralization pattern and main cluster in the right MCA). ACA, anterior cerebral artery; CAA, cerebral amyloid angiopathy; CMB, cerebral microbleeds; MCA, middle cerebral artery; PCA, posterior cerebral artery.

### 3.4 Sensitivity Analyses

When participants with a history of sICH are included in the study, the spatial clustering of CMBs across flow territories remained significant (*n* = 85; observed *χ* ^2^ = 102,027; mean permutated null distributions *χ* ^2^ = 5906 (SD = 838); Figure S2B). Inter‐rater reliability between the two raters was excellent for the subset of participants without sICH (ICC (2, 1) = 0.999, 95% CI [0.999, 1.000], F(28, 9.13) = 265,312) and for the total sample (ICC (2, 1) = 0.999, 95% CI [0.999, 1.000], F(70, 37.4) = 26,639).

Finally, tests to check robustness, by omitting any one or two covariate groups from the analyses, showed statistical significance for each combination of omitted covariate groups.

## 4 Discussion

Preferential clustering of CMBs was found in participants with (D‐)CAA without sICH. The effect remained robust when including participants with a history of sICH. In addition, on group level, the highest CMB densities were observed in the right PCA, right MCA, and left PCA. No lateralization pattern was evident when comparing densities across matched flow territories.

These findings extend prior evidence of lobar‐level CMB clustering in CAA by focusing on an alternative vascular anatomical perspective with increased power [7]. Furthermore, by separately analyzing patients with and without ICH, it was demonstrated that the observed clustering is not primarily driven by ICH‐related effects.

The fact that clustering within flow territories was observed may indicate that pathology, such as vascular inflammation, or physiology, such as increased pulsatility, affects a certain part of the vasculature, thereby increasing the risk for CMBs in that territory. On the other hand, these findings do not exclude the possibility of clustering independent of the underlying vasculature, for example, through local spreading of inflammation or local disruption of homeostasis after an event such as a CMB. More generally, due to the cross‐sectional nature of the data, it could not be identified whether the clustering we observed is due to (1) underlying spatial variation in CAA pathology across vascular territories, or (2) temporal correlations, for example a progressive weakening of a region following prior hemorrhages and thus later CMBs preferentially occurring in the neighborhood of earlier ones. This is the result of the well‐known phenomenon of clustering‐intensity confounding [26]. Therefore, our results should be interpreted as evidence of clustering without specification of its origin.

In this study, arterial flow territories were chosen as regions of interest rather than purely anatomical lobes. This choice was based on the notion that the primary pathology of CAA, amyloid‐β deposition, affects the arterial vasculature, and that vascular factors such as vessel pulsatility and reactivity may vary and thus contribute to local vulnerability [5, 27, 28]. In addition, prior work has shown multiple CAA‐related markers, CMB, ICH, and PET Aβ tracer binding, do not have a strictly lobar predilection. Research has shown that CMBs in CAA are not confined to a single lobe but exhibit a broader posterior predilection, mainly in the occipital and temporal lobes, while ICHs and Aβ deposition on pathology and PET likewise follow vascular, rather than strictly anatomical patterns [7, 11, 29].

The finding of individual variations in regional vulnerability may reflect personalized disease trajectories or focal triggers. While individual CMBs are generally clinically silent, severe localized clustering within distinct flow territories may help explain the wide clinical heterogeneity observed among patients with CAA and similar total lesion burdens. For instance, asymmetric microvascular damage within specific arterial networks could theoretically account for domain‐specific phenotypes, such as asymmetric cognitive decline. In addition, the interindividual variability is not limited to the non‐sICH subset, indicating that the finding is not influenced by the introduction of large disruptive pathology that disturbs the CMB distribution.

The highest median CMB densities in the right and left PCA and right MCA territories align with previous literature revealing a posterior dominance of CAA‐related pathology, not limited to the occipital lobe but extending into the temporal and parietal lobe [7]. Similar posterior lobes‐predilection of CMBs was seen in the general population and in patients with Alzheimer's Disease [30, 31]. Although on the individual level clear left/right differences can be observed as a reflection of their tendency to cluster together, on a group level, a preferential left or right location for CMBs was not observed.

Strengths of the study include the large D‐CAA and sCAA sample with a high total number of CMBs leading to high statistical power. In addition, imaging protocols were harmonized throughout the participants, and the main scoring was done by one trained observer, which assures homogenous sensitivity to CMBs. Through permutation‐based inference, the significance of our findings can robustly be assessed, and the unweighted chi‐square test provided an appropriate assessment of the data's large, sparse contingency table. In addition, our sensitivity analyses of omitting any one or two covariate groups per analysis, showed robustness of our results. Indicating that the results were not driven by a few participants with atypical distributions. Also, manual CMB annotations ensured high accuracy even with large numbers and dense clusters of CMBs.

## 5 Limitations

Limitations to the current study are, first, the use of a flow territory atlas instead of individual flow territory mapping. This may result in inaccuracies, especially in the presence of individual flow territory abnormalities, variations of the Circle of Willis, or variations in the flow territory border zones between participants [32]. However, given the large sample size and high number of total CMBs, any resulting bias is expected to be non‐significant. Second, local effects of ICH may influence the number of regional CMBs in two ways: firstly, by reducing the amount of viable parenchyma in and around the hematoma region (leaving less tissue in which CMBs can occur). Secondly, ICHs may create a locally pro‐hemorrhagic environment vulnerability due to perihematomal neuroinflammation with microglial/astrocytic activation and sustained blood–brain barrier dysfunction driven by thrombin, hemoglobin, and iron, which could increase susceptibility to new microbleeds in adjacent tissue [33, 34]. These well‐described mechanisms motivated the exclusion of patients with sICH in the primary analysis. However, the robustness of the findings in both samples, that is, including and excluding participants with a history of sICH, indicates that local effects of sICH are not the primary driver of the results. A further limitation is the relatively high proportion of participants classified as smokers, particularly in the sCAA cohort. This likely reflects the broad definition of smoking (ever vs. never), which may overestimate current or clinically relevant exposure. The substantial baseline age difference between the sCAA and D‐CAA cohorts is acknowledged and was expected. While advanced age drives sCAA pathology, D‐CAA features an accelerated disease course manifesting from a significantly younger age. Since the age of disease onset differs greatly between these two CAA subtypes, this cross‐group difference is an expected demographic characteristic. In this analysis, the potential effect of age is accounted for by evaluating relative intra‐individual distributions within age‐ and covariate‐matched strata. Furthermore, healthy age‐matched controls were not included in this study. Because healthy individuals rarely present with multiple lobar CMBs, which means that they lack the necessary data density required to compute meaningful observed‐versus‐expected territorial distributions or to undergo permutation testing. It is also noted that cardiovascular risk factors, such as hypertension and hypercholesterolemia, were more prevalent in the sCAA cohort than the D‐CAA cohort. Where important risk factors of microvascular disease, specifically hypertension and smoking status, were explicitly controlled for in the exact‐matching covariate design, hypercholesterolemia was omitted. Including all recorded vascular risk factors as matching criteria would over‐stratify the sample, resulting in subgroups too small to yield meaningful statistical distributions. However, while unmatched risk factors could potentially influence absolute CMB counts between the cohorts, they do not impact the main finding of individual spatial clustering, which remains robust within the matched strata. Another limitation is the relatively high number of CMBs per participant observed in this cohort compared to other published series [35, 36, 37]. This is likely explained by two factors: first, the high quality of the 3 T SWI protocol, which enables sensitive detection of even small or clustered CMBs; and second, the fact that the center where this study was performed, functions as a national referral site for CAA, which may bias the study population toward patients with more advanced disease. While this, together with single center, vendor, field strength, and scanner acquisition, may limit the generalizability of the absolute CMB counts to other cohorts, it is not expected to substantially affect the main findings, as the observed clustering patterns are relative within individuals—with a large variability in CMB counts—and thus unlikely to be driven by the overall CMB burden. Lastly, although we observe an excellent inter‐rater reliability, it is important to note that this measure is limited to the assessment of total CMB count, but provides no information about whether the exact same CMBs are identified.

## 6 Conclusion

CMBs in CAA tend to cluster on an individual level. These findings suggest variable regional vulnerability beyond known group‐level trends, which may be induced by localized CAA pathology and/or resulting inflammation. Given the cross‐sectional nature of this analysis, the causal drivers of the observed individual variations cannot be definitively determined. While localized disease progression remains a highly plausible mechanism, where an initial vascular or inflammatory event triggers a cascade of regional susceptibility, more data is needed to elucidate this hypothesis. For future work, it would be relevant to further look into the underlying mechanisms that are related to clustering of CMBs. For example, longitudinal studies, contrast‐enhanced MRI, and histopathological research may provide information on the processes related to inflammation, blood–brain barrier breakdown, and pathology related to the vascular tree. In addition, longitudinal data would allow assessment of spatial progression and temporal evolution of CMB patterns, as well as the relation of CMB patterns to the development of other CAA‐related MRI markers, in order to study the predictive value of local hemorrhagic vulnerability.

## Funding

The FOCAS and AURORA studies were financially supported by Dekker grant 2016T86 from the Dutch Heart Foundation.

## Conflicts of Interest

Manon R. Schipper reports independent support from the TRACK D‐CAA consortium, consisting of Alnylam, Biogen, the Dutch CAA foundation, Vereniging HCHWA‐D, and researchers from Leiden, Boston, and Perth. Reinier G. J. van der Zwet reports independent support from the TRACK D‐CAA consortium, consisting of Alnylam, Biogen, the Dutch CAA foundation, Vereniging HCHWA‐D, and researchers from Leiden, Boston, and Perth. Marieke J. H. Wermer reports independent support from the Dutch Research Council (NWO) ZonMw (VIDI grant 91717337) and the Netherlands Heart Foundation (Dekker grant 2016T86). Matthias J. P. van Osch reports support by a NWO‐Human Measurement Models 2.0 grant (18969) as well as support from the Dutch Research Council (NWO), a grant from the Leducq Foundation and the Leducq Foundation for Cardiovascular Research (23CVD03), and the Dutch Brain Foundation. Matthias J. P. van Osch is an unpaid member of the clinical trial steering committee of the cAPPricorn trial of Alnylam. The remaining authors declare no conflicts of interest.

## Supporting information


**Table S1:** Participants characteristics of all participants.
**Table S2:** Covariate subgroup sizes.
**Figure S1:** Flowchart of patient selection.
**Figure S2:** Histograms of test statistics under the null hypothesis, compared to the observed test statistic indicated with the vertical blue dashed line.

**File S1:** r code for statistical analysis copy for sharing revised.

**File S2:** STROBE Statement—checklist of items that should be included in reports of observational studies.
