# Supplementary material for: Clustering of Cerebral Microbleeds in Cerebral Amyloid Angiopathy: A Flow Territory‐Based Analysis
Source: J Magn Reson Imaging. 2026 Jul 25;64(5):1241–9. doi: 10.1002/jmri.70454 (PMC13667336; doi:10.1002/jmri.70454)
Supplement: Supplementary file 1 [file JMRI-64-1241-s001.docx]

STROBE Statement—checklist of items that should be included in reports of observational studies

|  | Item No. | Recommendation | Page  No. | Relevant text from manuscript |
| --- | --- | --- | --- | --- |
| **Title and abstract** | 1 | (*a*) Indicate the study’s design with a commonly used term in the title or the abstract |  |  |
|  |  | (*b*) Provide in the abstract an informative and balanced summary of what was done and what was found | 2 | Line 16- 45 |
| Introduction | | | |  |
| Background/rationale | 2 | Explain the scientific background and rationale for the investigation being reported | 4-5 | Line 52-81 |
| Objectives | 3 | State specific objectives, including any prespecified hypotheses | 5 | Line 82-86 |
| Methods | | | |  |
| Study design | 4 | Present key elements of study design early in the paper | 6 | Line 89-102 |
| Setting | 5 | Describe the setting, locations, and relevant dates, including periods of recruitment, exposure, follow-up, and data collection |  |  |
| Participants | 6 | *Cross-sectional study*—Give the eligibility criteria, and the sources and methods of selection of participants | 6 | Line 89-102 |
| Variables | 7 | Clearly define all outcomes, exposures, predictors, potential confounders, and effect modifiers. Give diagnostic criteria, if applicable | 6-8 | Line 89-102  Line 130-158  Line 161-164 |
| Data sources/ measurement | 8* | For each variable of interest, give sources of data and details of methods of assessment (measurement). Describe comparability of assessment methods if there is more than one group | 6-7 | Line 98-100  Line 104-128 |
| Bias | 9 | Describe any efforts to address potential sources of bias | 6-8 | Line 96-98  Line 130-135  Line 159-160 |
| Study size | 10 | Explain how the study size was arrived at | 6 | Line 91 |

Continued on next page

| Quantitative variables | 11 | Explain how quantitative variables were handled in the analyses. If applicable, describe which groupings were chosen and why | 7-8 | Line 130-164 |
| --- | --- | --- | --- | --- |
| Statistical methods | 12 | (*a*) Describe all statistical methods, including those used to control for confounding | 7-8 | Line 130-70 |
|  |  | (*b*) Describe any methods used to examine subgroups and interactions | n.a. | n.a. |
|  |  | (*c*) Explain how missing data were addressed | n.a. | n.a. |
|  |  | (*d*) *Cross-sectional study*—If applicable, describe analytical methods taking account of sampling strategy | n.a. | n.a. |
|  |  | (*e*) Describe any sensitivity analyses | 7 | Line 159-160 |
| Results | | | | |
| Participants | 13* | (a) Report numbers of individuals at each stage of study—eg numbers potentially eligible, examined for eligibility, confirmed eligible, included in the study, completing follow-up, and analysed | Supplemental Figure 1 | Supplemental Figure 1 |
|  |  | (b) Give reasons for non-participation at each stage | Supplemental Figure 1 | Supplemental Figure 1 |
|  |  | (c) Consider use of a flow diagram | Supplemental Figure 1 | Supplemental Figure 1 |
| Descriptive data | 14* | (a) Give characteristics of study participants (eg demographic, clinical, social) and information on exposures and potential confounders | Table 1  Supplementary Table 1 | Table 1  Supplementary Table 1 |
|  |  | (b) Indicate number of participants with missing data for each variable of interest | n.a. | n.a. |
| Outcome data | 15* | *Cross-sectional study—*Report numbers of outcome events or summary measures | 9  12 | Line 176-177  Line 247-248 |
| Main results | 16 | (*a*) Give unadjusted estimates and, if applicable, confounder-adjusted estimates and their precision (eg, 95% confidence interval). Make clear which confounders were adjusted for and why they were included | n.a. | n.a. |
|  |  | (*b*) Report category boundaries when continuous variables were categorized | n.a. | n.a. |
|  |  | (*c*) If relevant, consider translating estimates of relative risk into absolute risk for a meaningful time period | n.a. | n.a. |

Continued on next page

| Other analyses | 17 | Report other analyses done—eg analyses of subgroups and interactions, and sensitivity analyses | 9-10 | Line 185-190  Line 203-206 |
| --- | --- | --- | --- | --- |
| Discussion | | | | |
| Key results | 18 | Summarise key results with reference to study objectives | 11 | Line 208-211 |
| Limitations | 19 | Discuss limitations of the study, taking into account sources of potential bias or imprecision. Discuss both direction and magnitude of any potential bias | 11-12 | Line 256-280 |
| Interpretation | 20 | Give a cautious overall interpretation of results considering objectives, limitations, multiplicity of analyses, results from similar studies, and other relevant evidence | 11-12 | Line 217-246 |
| Generalisability | 21 | Discuss the generalisability (external validity) of the study results | 13 | Line 277-283 |
| Other information | |  | | |
| Funding | 22 | Give the source of funding and the role of the funders for the present study and, if applicable, for the original study on which the present article is based | 14 | Line 293-312 |

*Give information separately for cases and controls in case-control studies and, if applicable, for exposed and unexposed groups in cohort and cross-sectional studies.

**Note:** An Explanation and Elaboration article discusses each checklist item and gives methodological background and published examples of transparent reporting. The STROBE checklist is best used in conjunction with this article (freely available on the Web sites of PLoS Medicine at http://www.plosmedicine.org/, Annals of Internal Medicine at http://www.annals.org/, and Epidemiology at http://www.epidem.com/). Information on the STROBE Initiative is available at www.strobe-statement.org.
