# Supplementary material for: Clustering of Cerebral Microbleeds in Cerebral Amyloid Angiopathy: A Flow Territory‐Based Analysis
Source: J Magn Reson Imaging. 2026 Jul 25;64(5):1241–9. doi: 10.1002/jmri.70454 (PMC13667336; doi:10.1002/jmri.70454)
Supplement: Supplementary file 2 [file JMRI-64-1241-s002.docx]

**Supplementary Materials**

Table S1*.* Participants characteristics of all participants

| Characteristics | All participants (n = 85) | D-CAA (n = 41) | sCAA (n = 44) |
| --- | --- | --- | --- |
| Age in years, mean (SD) | 64.9 (10.8) | 56.8 (7.7) | 72.5 (7.1) |
| Female sex | 36 (42%) | 20 (49%) | 16 (36%) |
| Hypertension | 42 (49%) | 12 (29%) | 30 (68%) |
| Hypercholesterolemia | 41 (48%) | 13 (32%) | 28 (64%) |
| Diabetes mellitus type 2 | 5 (6%) | 2 (5%) | 3 (7%) |
| Smoking, ever | 68 (80%) | 28 (68%) | 40 (91%) |
| History of ICH | 52 (61%) | 27 (66%) | 25 (57%) |
| Total CMB, median (IQR) | 107 (33 – 295) | 145 (72 – 377) | 66 (28 – 201) |
| Cerebellar CMB, median (IQR) | 2 (0 – 8) | 3 (0 – 17) | 2 (0 – 4) |

*Abbreviations.* D-CAA; Dutch-type Cerebral Amyloid Angiopathy. sCAA; Sporadic Cerebral Amyloid Angiopathy. sICH; symptomatic intracerebral hemorrhage. CMB; cerebral microbleeds.

Table S2*.* Covariate subgroup sizes

|  | All participants  (n = 85) | Participants without sICH (n =33) |
| --- | --- | --- |
| Covariate subgroups (disease type: hypertension status: smoking status: sex: binned age) |  |  |
| D-CAA:hypt_no:smok_no:male:50 | 2 | N/A |
| D-CAA:hypt_no:smok_no:male:60 | 3 | N/A |
| D-CAA:hypt_no:smok_no:female:50 | 1 | 1 |
| D-CAA:hypt_no:smok_no:female:60 | 2 | 1 |
| D-CAA:hypt_no:smok_no:female:80 | 1 | N/A |
| D-CAA:hypt_no:smok_yes:male:40 | 2 | 2 |
| D-CAA:hypt_no:smok_yes:male:50 | 3 | N/A |
| D-CAA:hypt_no:smok_yes:male:60 | 7 | N/A |
| D-CAA:hypt_no:smok_yes:female:40 | 1 | 1 |
| D-CAA:hypt_no:smok_yes:female:50 | 4 | 2 |
| D-CAA:hypt_no:smok_yes:female:60 | 1 | 1 |
| D-CAA:hypt_no:smok_yes:female:70 | 2 | 1 |
| D-CAA:hypt_yes:smok_no:male:60 | 1 | N/A |
| D-CAA:hypt_yes:smok_no:male:70 | 1 | 1 |
| D-CAA:hypt_yes:smok_no:female:50 | 1 | N/A |
| D-CAA:hypt_yes:smok_no:female:60 | 1 | 1 |
| D-CAA:hypt_yes:smok_yes:male:50 | 1 | 1 |
| D-CAA:hypt_yes:smok_yes:male:60 | 1 | 1 |
| D-CAA:hypt_yes:smok_yes:female:50 | 1 | N/A |
| D-CAA:hypt_yes:smok_yes:female:60 | 2 | N/A |
| D-CAA:hypt_yes:smok_yes:female:70 | 3 | 1 |
| sCAA:hypt_no:smok_no:female:70 | 1 | N/A |
| sCAA:hypt_no:smok_yes:male:60 | 2 | 1 |
| sCAA:hypt_no:smok_yes:male:70 | 3 | 2 |
| sCAA:hypt_no:smok_yes:male:80 | 2 | 1 |
| sCAA:hypt_no:smok_yes:female:60 | 2 | 1 |
| sCAA:hypt_no:smok_yes:female:70 | 3 | 2 |
| sCAA:hypt_no:smok_yes:female:80 | 1 | N/A |
| sCAA:hypt_yes:smok_no:male:70 | 2 | N/A |
| sCAA:hypt_yes:smok_no:male:80 | 1 | N/A |
| sCAA:hypt_yes:smok_yes:male:60 | 3 | N/A |
| sCAA:hypt_yes:smok_yes:male:70 | 10 | 4 |
| sCAA:hypt_yes:smok_yes:male:80 | 3 | 2 |
| sCAA:hypt_yes:smok_yes:male:90 | 2 | 1 |
| sCAA:hypt_yes:smok_yes:female:60 | 2 | 1 |
| sCAA:hypt_yes:smok_yes:female:70 | 1 | N/A |
| sCAA:hypt_yes:smok_yes:female:80 | 6 | 4 |

*Abbreviations.* D-CAA; Dutch-type Cerebral Amyloid Angiopathy. sCAA; Sporadic Cerebral Amyloid Angiopathy. sICH; symptomatic intracerebral hemorrhage. N/A; not applicable.

*Note.* Age is presented in bins of 10 years.

**Figure S1.** Flowchart of patient selection

*Abbreviations.* CAA-ri; Cerebral Amyloid Angiopathy related inflammation. sCAA; sporadic Cerebral Amyloid Angiopathy. D-CAA; Dutch-type Cerebral Amyloid Angiopathy. sICH; symptomatic intracerebral hemorrhage.

**Figure S2.** Histograms of test statistics under the null hypothesis, compared to the observed test statistic indicated with the vertical blue dashed line.

*Abbreviations.* sICH; symptomatic intracerebral hemorrhage.
