# Supplementary material for: Clustering of Cerebral Microbleeds in Cerebral Amyloid Angiopathy: A Flow Territory‐Based Analysis
Source: J Magn Reson Imaging. 2026 Jul 25;64(5):1241–9. doi: 10.1002/jmri.70454 (PMC13667336; doi:10.1002/jmri.70454)
Supplement: Supplementary file 3 [file JMRI-64-1241-s003.docx]

\# -----------------------------------------------------------------

\# Script by Jelle Goeman, Manon Schipper and Reinier van der Zwet

\# Used for version 7 of the manuscript

\# Last updated: 30-06-2026

\# -----------------------------------------------------------------

\# -----------------------------------------------------------------

\# README: Requirements to run this script

\# -----------------------------------------------------------------

\#

\# Required data files (must be placed in data/ directory):

\# 1. Lokatie_bloedingen_AURORA_FOCAS_baseline_flowterritory_covariates_inclvolumes.xls --> main dataframe

\# 2. listofsympICH.sav --> ICH status

\#

\# Required R packages (automatically installed and loaded below):

\# tidyverse, ggplot2, readxl, haven, kableExtra, ggbreak, scales, patchwork

\#

\# Null Distribution System:

\# - By default, the script loads pre-computed null distributions for reproducibility

\# - To generate new distributions: Uncomment 'REGENERATE_NULL_DISTRIBUTIONS <- TRUE'

\# - Distributions are saved in data/null_distributions/ for future use

\#

\# Output: Creates HTML tables and PNG figures in output/ directory and test statistics in console

\# -----------------------------------------------------------------

\# -----------------------------------------------------------------

\# Install and load libraries

\# -----------------------------------------------------------------

\# Uncomment the following lines to install the packages if needed

#install.packages("tidyverse")

#install.packages("ggplot2")

#install.packages("readxl")

#install.packages("haven")

#install.packages("kableExtra")

#install.packages("ggbreak")

#install.packages("scales")

#install.packages("patchwork")

library(tidyverse)

library(ggplot2)

library(readxl)

library(haven)

library(kableExtra)

library(ggbreak)

library(scales)

library(patchwork)

\# -----------------------------------------------------------------

\# Import and clean data

\# -----------------------------------------------------------------

\# Import main dataframe

D <- read_xls("data/Lokatie_bloedingen_AURORA_FOCAS_baseline_flowterritory_covariates_inclvolumes.xls")

\# remove macro, removes empty "unknown" variable and "Column1"(=sum of bleeds):

D <- D %>% filter(type != "macro") %>% select(-unknown, -Column1)

\# Update missing smoking values

D <- D %>%

mutate(smoking = case_when(

grepl("^PATIENT_ID_5", patient) ~ 0,

TRUE ~ smoking # Keep existing values for other patients

))

\# remove patients with missing smoking and mutstat values

D <- D %>% filter(mutstat != 9999) %>% filter(smoking != 9999) %>% filter(!is.na(smoking))

\# Exclude patients with history of CAA-ri

D <- D %>% filter(!(patient %in% c("PATIENT_ID_1", "PATIENT_ID_2")))

#RvdZ 09-07-2025: Exclude patient with erroneous CMB counts

#PATIENT_ID_3: According to mask file and MicroMesoMacroMasks database CMB count = 24, but our data shows 6.

#PATIENT_ID_4: No mask available, SWI shows at least 4 CMBs according to MvW, but our data shows 1 CMB.

D <- D %>% filter(!(patient %in% c("PATIENT_ID_3", "PATIENT_ID_4")))

\# Import SPSS file containing ICH information

ich_data <- read_sav("data/listofsympICH.sav")

\# Filter for patients with ICH (ICH == 1) and select their IDs

patients_to_exclude_ich <- ich_data %>%

filter(ICH == 1) %>%

pull(ID) # Assumes the ID column is named 'ID'

\# Create the new dataset D_no_ICH by filtering the original D dataframe

D_no_ICH <- D %>% filter(!(patient %in% patients_to_exclude_ich))

\# collapse mutstat

D <- D %>% mutate(mutstat = fct_collapse(factor(mutstat), aurora=c("1","2"), focas="3"))

\# turn all into factor

D <- D %>% mutate(hypertensie = factor(hypertensie, labels = c("hypt_no", "hypt_yes")),

smoking=factor(smoking, labels = c("smok_no", "smok_yes")),

sex=factor(sex, labels=c("male", "female")))

\# long format:

counts <- D %>% pivot_longer(LeftproximalACA:RightdistalPCA, names_to = "region",

values_to = "n")

\# First: Pool 18 regions into 6 regions (remove "distal", "middle" & "proximal")

\# Get all non-region, non-count, non-patient variable names dynamically

patient_vars <- counts %>% select(-region, -n, -patient) %>% names()

counts <- counts %>%

mutate(area = str_remove(region, "distal")) %>%

mutate(area = str_remove(area, "proximal")) %>%

mutate(area = str_remove(area, "middle")) %>%

mutate(area = as.factor(area)) %>%

select(-region) %>%

group_by(patient, area) %>%

summarise(

n = sum(n),

across(all_of(patient_vars), first),

.groups = "drop"

)

\# Load cerebellar data

cerebellar_data <- read_excel("data/cerebellar_bleeds.xlsx", sheet = "data", range = "A1:G1000") %>%

select(StudyID, removed_Right_Cerebellum, removed_Left_Cerebellum) %>%

rename(patient = StudyID) %>%

filter(!is.na(patient)) %>%

filter(!is.na(removed_Right_Cerebellum)) %>%

filter(!is.na(removed_Left_Cerebellum))

\# Second: Apply cerebellar correction to the 6-region data

counts <- counts %>%

left_join(cerebellar_data, by = "patient") %>%

mutate(

removed_Right_Cerebellum = ifelse(is.na(removed_Right_Cerebellum), 0, removed_Right_Cerebellum),

removed_Left_Cerebellum = ifelse(is.na(removed_Left_Cerebellum), 0, removed_Left_Cerebellum),

n = case_when(

area == "RightPCA" ~ pmax(0, n - removed_Right_Cerebellum),

area == "LeftPCA" ~ pmax(0, n - removed_Left_Cerebellum),

TRUE ~ n

)

) %>%

select(-removed_Right_Cerebellum, -removed_Left_Cerebellum)

#Sums micro and mesobleeds to determine total number of CMB per patient (after cerebellar correction)

patient_total <- counts %>% group_by(patient) %>% summarize(total=sum(n))

#remove patients with zero counts

counts <- counts %>% left_join(patient_total, by="patient") %>% filter(total != 0) %>% select(-total) %>%

mutate(patient=as.factor(patient))

\# Create counts subset excluding ICH patients

counts_no_ICH <- counts %>% filter(!(patient %in% patients_to_exclude_ich))

\# Create CMB density variable by dividing n CMBs by volume per region. This assigns NA to CMB_density if n = 0

\# Convert from per mm³ to per cm³ by multiplying by 1000

\# For whole group

counts_density_all <- counts %>%

mutate(

CMB_density = case_when(

n == 0 ~ n / volume_leftACA, # If counts$n is zero, assign a zero

n != 0 & area == "LeftACA" ~ (n / volume_leftACA) * 1000, # Else if area == "LeftACA", do this

n != 0 & area == "LeftMCA" ~ (n / volume_leftMCA) * 1000, # Else if area == "LeftMCA", do this, etc.

n != 0 & area == "LeftPCA" ~ (n / volume_leftPCA) * 1000,

n != 0 & area == "RightACA" ~ (n / volume_rightACA) * 1000,

n != 0 & area == "RightMCA" ~ (n / volume_rightMCA) * 1000,

n != 0 & area == "RightPCA" ~ (n / volume_rightPCA) * 1000,

TRUE ~ NA_real_ # If none of the above conditions match, assign NA

)

)

\# Create CMB density variable by dividing n CMBs by volume per region. This assigns NA to CMB_density if n = 0

\# Convert from per mm³ to per cm³ by multiplying by 1000

\# For no sICH subgroup

counts_density <- counts_no_ICH %>%

mutate(

CMB_density = case_when(

n == 0 ~ n / volume_leftACA, # If counts$n is zero, assign a zero

n != 0 & area == "LeftACA" ~ (n / volume_leftACA) * 1000, # Else if area == "LeftACA", do this

n != 0 & area == "LeftMCA" ~ (n / volume_leftMCA) * 1000, # Else if area == "LeftMCA", do this, etc.

n != 0 & area == "LeftPCA" ~ (n / volume_leftPCA) * 1000,

n != 0 & area == "RightACA" ~ (n / volume_rightACA) * 1000,

n != 0 & area == "RightMCA" ~ (n / volume_rightMCA) * 1000,

n != 0 & area == "RightPCA" ~ (n / volume_rightPCA) * 1000,

TRUE ~ NA_real_ # If none of the above conditions match, assign NA

)

)

\# get out the covariates, create rounded age variable

covars <- counts %>% select(-type, -area, -n) %>% distinct() %>%

mutate(age10 = factor(round(age, -1)))

\# Join ICH status into covars dataframe

ich_status_for_covars <- ich_data %>%

select(ID, ICH) %>%

rename(patient = ID)

covars <- covars %>%

left_join(ich_status_for_covars, by = "patient") %>%

mutate(ICH = ifelse(is.na(ICH), 0, ICH)) # Assume NA means no ICH history (0), since the listofsympICH is only those with ICH

\# Join total CMB counts into covars

covars <- covars %>%

left_join(patient_total, by = "patient") # Adds the 'total' column

\# -----------------------------------------------------------------

\# Calculate total CMB counts by group (sCAA vs D-CAA)

\# -----------------------------------------------------------------

\# Calculate total CMBs for all patients

total_CMBs_all_patients <- sum(covars$total)

total_CMBs_sCAA_all <- sum(covars$total[covars$mutstat == "focas"])

total_CMBs_DCAA_all <- sum(covars$total[covars$mutstat == "aurora"])

\# Calculate total CMBs for no ICH patients

covars_no_ICH <- covars %>% filter(!(patient %in% patients_to_exclude_ich))

total_CMBs_all_noICH <- sum(covars_no_ICH$total)

total_CMBs_sCAA_noICH <- sum(covars_no_ICH$total[covars_no_ICH$mutstat == "focas"])

total_CMBs_DCAA_noICH <- sum(covars_no_ICH$total[covars_no_ICH$mutstat == "aurora"])

\# Report results to console

print("=================================================================")

print("TOTAL CMB COUNTS BY GROUP")

print("=================================================================")

print(paste0("ALL PATIENTS:"))

print(paste0(" Total CMBs across all patients: ", total_CMBs_all_patients))

print(paste0(" Total CMBs in sCAA patients: ", total_CMBs_sCAA_all))

print(paste0(" Total CMBs in D-CAA patients: ", total_CMBs_DCAA_all))

print("")

print(paste0("NO ICH PATIENTS:"))

print(paste0(" Total CMBs across all no ICH patients: ", total_CMBs_all_noICH))

print(paste0(" Total CMBs in sCAA no ICH patients: ", total_CMBs_sCAA_noICH))

print(paste0(" Total CMBs in D-CAA no ICH patients: ", total_CMBs_DCAA_noICH))

print("=================================================================")

\# Cerebellar counts no ICH

covars_no_ICH_cerebellar <- covars_no_ICH %>%

left_join(cerebellar_data, by = "patient") %>%

mutate(

removed_Right_Cerebellum = ifelse(is.na(removed_Right_Cerebellum), 0, removed_Right_Cerebellum),

removed_Left_Cerebellum = ifelse(is.na(removed_Left_Cerebellum), 0, removed_Left_Cerebellum),

)

covars_no_ICH_cerebellar$total_cerebellar <- covars_no_ICH_cerebellar$removed_Right_Cerebellum + covars_no_ICH_cerebellar$removed_Left_Cerebellum

summary(covars_no_ICH_cerebellar$total_cerebellar)

summary(covars_no_ICH_cerebellar$total_cerebellar[covars_no_ICH_cerebellar$mutstat == "focas"])

summary(covars_no_ICH_cerebellar$total_cerebellar[covars_no_ICH_cerebellar$mutstat == "aurora"])

\# Cerebellar counts total sample

covars_cerebellar <- covars %>%

left_join(cerebellar_data, by = "patient") %>%

mutate(

removed_Right_Cerebellum = ifelse(is.na(removed_Right_Cerebellum), 0, removed_Right_Cerebellum),

removed_Left_Cerebellum = ifelse(is.na(removed_Left_Cerebellum), 0, removed_Left_Cerebellum),

)

covars_cerebellar$total_cerebellar <- covars_cerebellar$removed_Right_Cerebellum + covars_cerebellar$removed_Left_Cerebellum

summary(covars_cerebellar$total_cerebellar)

summary(covars_cerebellar$total_cerebellar[covars_cerebellar$mutstat == "focas"])

summary(covars_cerebellar$total_cerebellar[covars_cerebellar$mutstat == "aurora"])

\# -----------------------------------------------------------------

\# End of CMB totals calculation

\# -----------------------------------------------------------------

\# check and create distinct covariate combinations

covars %>% count(mutstat, hypertensie, smoking, age10, sex) %>% arrange(desc(n))

pattern <- covars %>% mutate(combination = mutstat:hypertensie:smoking:sex:age10) %>%

select(patient, combination)

\# expand counts

expandedcounts <- counts %>% uncount(n) %>% left_join(pattern, by="patient")

\# This is the line that shuffles the patients within each combination (which is used for the null distribution)

\# We ran it here as test to check whether the shuffling worked

expandedcounts_test <- expandedcounts %>% group_by(combination) %>% mutate(patient = sample(patient)) %>% ungroup

\# find expected counts under H0: no residual variation between patients

#total = total per covariate group

total <- expandedcounts %>% group_by(combination) %>% count() %>% ungroup

#area_margin = proportion of bleeds per area (per covariate combination)

area_margin <- expandedcounts %>% group_by(combination) %>% count(area) %>%

#ungroup %>%

right_join(total, by = "combination") %>%

mutate(fraction = n.x/n.y) %>%

select(-n.x, -n.y) %>%

pivot_wider(names_from = area, values_from = fraction, values_fill = 0)

#expected = number of bleeds per region according to their covariate group: proportion times the total number of bleeds of this patient

expected <- expandedcounts %>% count(patient) %>%

left_join(pattern, by = "patient") %>% left_join(area_margin, by = "combination") %>%

pivot_longer(LeftACA:RightPCA, names_to = "area", values_to = "fraction") %>%

mutate(expected = n*fraction) %>%

select(-n, -fraction)

\# -----------------------------------------------------------------

\# PREPARE NO ICH SUBSET DATA (needed for null distribution loading/validation)

\# -----------------------------------------------------------------

\# Calculate expected counts for NO ICH subset

expandedcounts_no_ICH <- expandedcounts %>%

filter(patient %in% D_no_ICH$patient)

total_no_ICH <- expandedcounts_no_ICH %>% group_by(combination) %>% count() %>% ungroup %>% rename(n_combination = n)

\# Handle combinations that might only exist in full set but not subset

\# Also handle areas that might have zero counts within a combination in the subset

area_margin_no_ICH <- expandedcounts_no_ICH %>%

group_by(combination) %>%

count(area) %>%

ungroup() %>%

complete(combination, area, fill = list(n = 0)) %>%

left_join(total_no_ICH, by = "combination") %>%

mutate(fraction = ifelse(n_combination > 0, n / n_combination, 0)) %>%

select(combination, area, fraction) %>%

pivot_wider(names_from = area, values_from = fraction, values_fill = 0)

pattern_no_ICH <- expandedcounts_no_ICH %>% distinct(patient, combination)

expected_no_ICH <- expandedcounts_no_ICH %>%

count(patient) %>%

rename(n_patient_total = n) %>%

left_join(pattern_no_ICH, by = "patient") %>%

left_join(area_margin_no_ICH, by = "combination") %>%

pivot_longer(cols = matches("^(Left|Right)(ACA|MCA|PCA)$"),

names_to = "area",

values_to = "fraction") %>%

mutate(expected = n_patient_total * fraction) %>%

select(patient, area, expected, combination)

\# -----------------------------------------------------------------

\# Main analysis

\# -----------------------------------------------------------------

\# -----------------------------------------------------------------

\# NULL DISTRIBUTION CONTROL

\# Uncomment the following line to generate new null distributions

\# -----------------------------------------------------------------

REGENERATE_NULL_DISTRIBUTIONS <- FALSE

\# -----------------------------------------------------------------

\# NULL DISTRIBUTION MANAGEMENT FUNCTIONS

\# -----------------------------------------------------------------

\# Function to create null distributions directory

create_null_dist_directory <- function() {

null_dist_dir <- "data/null_distributions"

if (!dir.exists(null_dist_dir)) {

dir.create(null_dist_dir, recursive = TRUE)

print(paste("Created directory:", null_dist_dir))

}

return(null_dist_dir)

}

\# Function to generate data characteristics hash for validation

generate_data_hash <- function(expandedcounts, expandedcounts_no_ICH, expected, expected_no_ICH) {

\# Create a summary of key data characteristics

data_summary <- list(

n_patients_all = length(unique(expandedcounts$patient)),

n_patients_no_ICH = length(unique(expandedcounts_no_ICH$patient)),

n_bleeds_all = nrow(expandedcounts),

n_bleeds_no_ICH = nrow(expandedcounts_no_ICH),

n_combinations_all = length(unique(expandedcounts$combination)),

n_combinations_no_ICH = length(unique(expandedcounts_no_ICH$combination)),

expected_sum_all = round(sum(expected$expected), 2),

expected_sum_no_ICH = round(sum(expected_no_ICH$expected), 2)

)

\# Create simple hash from concatenated values

hash_string <- paste(unlist(data_summary), collapse = "_")

return(hash_string)

}

\# Function to save null distributions

save_null_distributions <- function(TTs, TTs_no_ICH, expandedcounts, expandedcounts_no_ICH, expected, expected_no_ICH) {

null_dist_dir <- create_null_dist_directory()

timestamp <- format(Sys.time(), "%Y%m%d_%H%M%S")

\# Save distributions

saveRDS(TTs, file.path(null_dist_dir, paste0("TTs_full_dataset_", timestamp, ".rds")))

saveRDS(TTs_no_ICH, file.path(null_dist_dir, paste0("TTs_no_ICH_", timestamp, ".rds")))

\# Create and save metadata

metadata <- data.frame(

timestamp = timestamp,

generation_date = Sys.time(),

n_patients_all = length(unique(expandedcounts$patient)),

n_patients_no_ICH = length(unique(expandedcounts_no_ICH$patient)),

n_permutations = length(TTs),

data_hash = generate_data_hash(expandedcounts, expandedcounts_no_ICH, expected, expected_no_ICH),

TTs_mean = round(mean(TTs), 2),

TTs_sd = round(sd(TTs), 2),

TTs_no_ICH_mean = round(mean(TTs_no_ICH), 2),

TTs_no_ICH_sd = round(sd(TTs_no_ICH), 2),

stringsAsFactors = FALSE

)

\# Save or update metadata file

metadata_file <- file.path(null_dist_dir, "distribution_metadata.csv")

if (file.exists(metadata_file)) {

existing_metadata <- read.csv(metadata_file, stringsAsFactors = FALSE)

updated_metadata <- rbind(existing_metadata, metadata)

} else {

updated_metadata <- metadata

}

write.csv(updated_metadata, metadata_file, row.names = FALSE)

print("=================================================================")

print("NULL DISTRIBUTIONS SAVED")

print("=================================================================")

print(paste("Full dataset distributions saved as: TTs_full_dataset_", timestamp, ".rds", sep=""))

print(paste("No ICH distributions saved as: TTs_no_ICH_", timestamp, ".rds", sep=""))

print(paste("Metadata updated in: distribution_metadata.csv"))

print("=================================================================")

return(metadata)

}

\# Function to load most recent null distributions

load_null_distributions <- function(expandedcounts, expandedcounts_no_ICH, expected, expected_no_ICH) {

null_dist_dir <- "data/null_distributions"

if (!dir.exists(null_dist_dir)) {

print("No null distributions directory found. Will generate new distributions.")

return(list(success = FALSE))

}

\# Find most recent files

full_files <- list.files(null_dist_dir, pattern = "TTs_full_dataset_.*\\.rds$", full.names = TRUE)

no_ich_files <- list.files(null_dist_dir, pattern = "TTs_no_ICH_.*\\.rds$", full.names = TRUE)

if (length(full_files) == 0 || length(no_ich_files) == 0) {

print("No existing null distribution files found. Will generate new distributions.")

return(list(success = FALSE))

}

\# Get most recent files (assuming timestamp format allows proper sorting)

latest_full_file <- full_files[which.max(file.mtime(full_files))]

latest_no_ich_file <- no_ich_files[which.max(file.mtime(no_ich_files))]

\# Load distributions

TTs <- readRDS(latest_full_file)

TTs_no_ICH <- readRDS(latest_no_ich_file)

\# Validate against current data

current_hash <- generate_data_hash(expandedcounts, expandedcounts_no_ICH, expected, expected_no_ICH)

\# Check metadata if available

metadata_file <- file.path(null_dist_dir, "distribution_metadata.csv")

if (file.exists(metadata_file)) {

metadata <- read.csv(metadata_file, stringsAsFactors = FALSE)

latest_metadata <- metadata[nrow(metadata), ] # Get most recent row

if (latest_metadata$data_hash != current_hash) {

print("WARNING: Current data characteristics differ from saved distributions!")

print("Consider regenerating null distributions by uncommenting REGENERATE_NULL_DISTRIBUTIONS <- TRUE")

print(paste("Saved hash:", latest_metadata$data_hash))

print(paste("Current hash:", current_hash))

}

}

print("=================================================================")

print("NULL DISTRIBUTIONS LOADED")

print("=================================================================")

print(paste("Loaded full dataset distributions from:", basename(latest_full_file)))

print(paste("Loaded no ICH distributions from:", basename(latest_no_ich_file)))

print(paste("Full dataset - Mean:", round(mean(TTs), 2), "SD:", round(sd(TTs), 2)))

print(paste("No ICH dataset - Mean:", round(mean(TTs_no_ICH), 2), "SD:", round(sd(TTs_no_ICH), 2)))

print("=================================================================")

return(list(success = TRUE, TTs = TTs, TTs_no_ICH = TTs_no_ICH))

}

\# Unweighted chi square for said H0

#Observed = bleeds per region per participant

calculate_chi_square <- function(expandedcounts, expected) {

observed <- expandedcounts %>% count(patient, area, .drop=FALSE) %>% rename(observed=n)

OE <- observed %>% left_join(expected, by = c("patient", "area")) %>%

mutate(diff2 = (observed - expected)^2)

test_statistic <- OE %>% summarise(sum(diff2)) %>% unlist

return(test_statistic)

}

\# -----------------------------------------------------------------

\# GENERATE OR LOAD NULL DISTRIBUTIONS

\# -----------------------------------------------------------------

\# Calculate observed test statistics

TT <- calculate_chi_square(expandedcounts, expected)

\# Check if we should regenerate distributions or load existing ones

if (exists("REGENERATE_NULL_DISTRIBUTIONS") && REGENERATE_NULL_DISTRIBUTIONS) {

print("Generating new null distributions...")

\# Generate null distribution for full dataset

TTs <- replicate(9999, {

shuffled <- expandedcounts %>% group_by(combination) %>%

mutate(patient = sample(patient)) %>% ungroup

calculate_chi_square(shuffled, expected)

})

\# Generate null distribution for no ICH dataset (we'll need this later)

TTs_no_ICH <- replicate(9999, {

shuffled_no_ICH <- expandedcounts_no_ICH %>% group_by(combination) %>%

mutate(patient = sample(patient)) %>% ungroup

calculate_chi_square(shuffled_no_ICH, expected_no_ICH)

})

\# Save both distributions

save_null_distributions(TTs, TTs_no_ICH, expandedcounts, expandedcounts_no_ICH, expected, expected_no_ICH)

} else {

\# Try to load existing distributions

load_result <- load_null_distributions(expandedcounts, expandedcounts_no_ICH, expected, expected_no_ICH)

if (load_result$success) {

TTs <- load_result$TTs

TTs_no_ICH <- load_result$TTs_no_ICH

} else {

\# Fallback: generate new distributions if loading failed

print("Fallback: Generating new null distributions...")

TTs <- replicate(9999, {

shuffled <- expandedcounts %>% group_by(combination) %>%

mutate(patient = sample(patient)) %>% ungroup

calculate_chi_square(shuffled, expected)

})

TTs_no_ICH <- replicate(9999, {

shuffled_no_ICH <- expandedcounts_no_ICH %>% group_by(combination) %>%

mutate(patient = sample(patient)) %>% ungroup

calculate_chi_square(shuffled_no_ICH, expected_no_ICH)

})

\# Save the newly generated distributions

save_null_distributions(TTs, TTs_no_ICH, expandedcounts, expandedcounts_no_ICH, expected, expected_no_ICH)

}

}

\# Calculate p-value for full dataset

p_value <- mean(c(TTs, TT) >= TT)

print("=================================================================")

print("FULL DATASET RESULTS")

print("=================================================================")

print(paste0("Mean permutation test statistic (all): ", mean(TTs)))

print(paste0("SD permutation test statistic (all): ", sd(TTs)))

print(paste0("p-value: ", sprintf("%.5f", p_value)))

print(paste0("Test statistic: ", TT))

print("=================================================================")

\# -----------------------------------------------------------------

\# NO ICH SUBSET ANALYSIS

\# (Note: TTs_no_ICH was already generated/loaded in the previous section)

\# (Note: expandedcounts_no_ICH and expected_no_ICH were prepared earlier)

\# -----------------------------------------------------------------

\# Calculate observed test statistic for NO ICH subset

TT_no_ICH <- calculate_chi_square(expandedcounts_no_ICH, expected_no_ICH)

\# Calculate p-value for NO ICH subset (using previously generated/loaded TTs_no_ICH)

p_value_no_ICH <- mean(c(TTs_no_ICH, TT_no_ICH) >= TT_no_ICH)

print("=================================================================")

print("NO ICH SUBSET RESULTS")

print("=================================================================")

print(paste0("Mean permutation test statistic (No ICH): ", mean(TTs_no_ICH)))

print(paste0("SD permutation test statistic (No ICH): ", sd(TTs_no_ICH)))

print(paste0("NO ICH subset - p-value: ", p_value_no_ICH))

print(paste0("NO ICH subset - Test statistic: ", TT_no_ICH))

print("=================================================================")

\# -----------------------------------------------------------------

\# Analysis for comparing CMB densities for the six flow territories

\# Non-parametric repeated measures Friedman test

\# -----------------------------------------------------------------

\# Mean table for quick overview of mean densities per region

median_table <- counts_density_all %>%

group_by(area) %>%

summarise(median_score = median(CMB_density)) %>%

arrange(area)

IQR_table <- counts_density_all %>%

group_by(area) %>%

summarise(IQR_score = IQR(CMB_density)) %>%

arrange(area)

quantile_table <- counts_density_all %>%

group_by(area) %>%

reframe(quantile_score = quantile(CMB_density)) %>%

arrange(area)

print(median_table)

print(IQR_table)

print(quantile_table)

\# Testing normality of the CMB densities

shapiro.test(counts_density_all$CMB_density)

\# Testing differences between regions

counts_density_all$patient <- as.character(counts_density_all$patient)

friedman.test(CMB_density ~ area | patient, data = counts_density_all)

\# Groupwise post-hoc testing

pairwise.wilcox.test(counts_density_all$CMB_density, counts_density_all$area,

paired = TRUE,

p.adjust.method = "holm") # or "bonferroni", "BH"

\# -----------------------------------------------------------------

\# Permutated p-values per leave-k-out covar group -- FOR JMRI REVISION

\# Function

\# -----------------------------------------------------------------

\# Calculates p-values by leaving out k covariate groups at a time.

\# For each combination of groups left out, it:

\#	1. Filters expandedcounts and expected to exclude those groups

\#	2. Generates a fresh null distribution (9999 permutations)

\#	3. Calculates the observed test statistic

\#	4. Returns p-values + sample sizes per left-out combination

run_leaveout_analysis <- function(expandedcounts,

expected,

pattern,

k = 1,

n_permutations = 9999) {

\# Get all unique covariate groups

all_groups <- unique(expandedcounts$combination)

\# Generate all combinations of k groups to leave out

leaveout_combos <- combn(all_groups, k, simplify = FALSE)

message(sprintf("Running leave-%d-out analysis: %d combinations to test...",

k, length(leaveout_combos)))

results <- purrr::map_dfr(leaveout_combos, function(groups_to_exclude) {

label <- paste(groups_to_exclude, collapse = " & ")

message(sprintf(" Processing: leaving out [ %s ]", label))

\# --- 1. Subset data to exclude the k groups ---

ec_sub <- expandedcounts %>% filter(!combination %in% groups_to_exclude)

exp_sub <- expected	%>% filter(!combination %in% groups_to_exclude)

\# --- 2. Observed test statistic for this subset ---

TTa <- calculate_chi_square(ec_sub, exp_sub)

\# --- 3. Generate null distribution (mirroring original approach) ---

TTs_sub <- replicate(n_permutations, {

shuffled <- ec_sub %>%

group_by(combination) %>%

mutate(patient = sample(patient)) %>%

ungroup()

calculate_chi_square(shuffled, exp_sub)

})

\# --- 4. P-value: proportion of null >= observed ---

p_value <- mean(c(TTs_sub, TTa) >= TTa)

\# --- 5. Sample size for the included groups ---

n <- pattern %>%

filter(!combination %in% groups_to_exclude) %>%

nrow()

tibble(

left_out	= label,

k_excluded	= k,

TTa	= TTa,

p_value	= p_value,

n	= n

)

})

return(results)

}

\# -----------------------------------------------------------------

\# Permutated p-values per leave-k-out covar group -- FOR JMRI REVISION

\# Run function

\# -----------------------------------------------------------------

\# Leave 1 covariate group out at a time

results_leaveout1 <- run_leaveout_analysis(

expandedcounts = expandedcounts,

expected	= expected,

pattern	= pattern,

k	= 1,

n_permutations = 9999

)

write.csv(results_leaveout1, "p_values_leave1out_9999.csv", row.names = FALSE)

\# Leave 2 covariate groups out at a time (k = 2)

results_leaveout2 <- run_leaveout_analysis(

expandedcounts = expandedcounts,

expected	= expected,

pattern	= pattern,

k	= 2,

n_permutations = 9999

)

write.csv(results_leaveout2, "p_values_leave2out_9999.csv", row.names = FALSE)

\# Leave 1 covariate groups out at a time — no ICH subgroup

results_leave1_no_ICH <- run_leaveout_analysis(

expandedcounts = expandedcounts_no_ICH,

expected	= expected_no_ICH,

pattern	= pattern_no_ICH,

k	= 1,

n_permutations = 9999

)

write.csv(results_leave1_no_ICH, "p_values_leave1out_no_ICH_9999.csv", row.names = FALSE)

\# Leave 2 covariate groups out at a time — no ICH subgroup (k = 2)

results_leave2_no_ICH <- run_leaveout_analysis(

expandedcounts = expandedcounts_no_ICH,

expected	= expected_no_ICH,

pattern	= pattern_no_ICH,

k	= 2,

n_permutations = 9999

)

write.csv(results_leave2_no_ICH, "p_values_leave2out_no_ICH_9999.csv", row.names = FALSE)

\# -----------------------------------------------------------------

\# Calculate ICC for JMRI revision

\# -----------------------------------------------------------------

\# Load CMB 2nd rater data

CMBICC_data <- read_sav("data/MicroMesoMacroMasks_2raters.sav") %>%

select(PatientID, MicroAmountMasks_r1, MicroAmountMasks_r2) %>%

rename(patient = PatientID) %>%

filter(MicroAmountMasks_r2 != 99999) %>%

filter(MicroAmountMasks_r1 != 99999) %>%

filter(!is.na(patient)) %>%

filter(!is.na(MicroAmountMasks_r1)) %>%

filter(!is.na(MicroAmountMasks_r2)) %>%

filter(MicroAmountMasks_r1 != 0)

CMBICC_data_no_ICH <- CMBICC_data %>% filter(!(patient %in% patients_to_exclude_ich))

\# install.packages("irr")

library(irr)

\# Calculate ICC

icc_data <- CMBICC_data[, c("MicroAmountMasks_r1", "MicroAmountMasks_r2")]

icc_results <- icc(icc_data, model = "twoway", type = "agreement", unit = "single")

icc_data_no_ICH <- CMBICC_data_no_ICH[, c("MicroAmountMasks_r1", "MicroAmountMasks_r2")]

icc_results_no_ICH <- icc(icc_data_no_ICH, model = "twoway", type = "agreement", unit = "single")

\# Print with more decimal places

print(icc_results$value, digits = 10)

print(icc_results_no_ICH$value, digits = 10)

\# Or look at the full object

str(icc_results)

str(icc_results_no_ICH)

\# -----------------------------------------------------------------

\# Table 1: Patient Characteristics

\# -----------------------------------------------------------------

\# Function to calculate n (%) for binary variables

binary_stats <- function(data, var_name) {

if(is.factor(data[[var_name]])) {

n_yes <- sum(data[[var_name]] == levels(data[[var_name]])[2])

} else {

n_yes <- sum(data[[var_name]] == 1)

}

pct_yes <- round(n_yes / nrow(data) * 100, 0)

return(paste0(n_yes, " (", pct_yes, "%)"))

}

\# Function to format age statistics

format_age <- function(data) {

paste0(round(mean(data$age), 1), " (", format(round(sd(data$age), 1), nsmall=1), ")")

}

\# Function to calculate and format Median (IQR)

format_median_iqr <- function(counts_vector) {

med <- round(median(counts_vector, na.rm = TRUE), 0)

q1 <- round(quantile(counts_vector, 0.25, na.rm = TRUE), 0)

q3 <- round(quantile(counts_vector, 0.75, na.rm = TRUE), 0)

return(paste0(med, " (", q1, " - ", q3, ")"))

}

\# Calculate statistics for each group

all_patients_covars <- covars

aurora_patients_covars <- covars %>% filter(mutstat == "aurora")

focas_patients_covars <- covars %>% filter(mutstat == "focas")

\# Create data frame for table

table1 <- data.frame(

Characteristic = c("Age, years", "Female sex", "Hypertension",

"Hypercholesterolemia", "Diabetes mellitus type 2",

"Smoking, ever", "History of ICH",

"Total CMB, median (IQR)"),

All = c(format_age(all_patients_covars),

binary_stats(all_patients_covars, "sex"),

binary_stats(all_patients_covars, "hypertensie"),

binary_stats(all_patients_covars, "hypercholesterolemia"),

binary_stats(all_patients_covars, "DM2"),

binary_stats(all_patients_covars, "smoking"),

binary_stats(all_patients_covars, "ICH"),

format_median_iqr(all_patients_covars$total)),

DCAA = c(format_age(aurora_patients_covars),

binary_stats(aurora_patients_covars, "sex"),

binary_stats(aurora_patients_covars, "hypertensie"),

binary_stats(aurora_patients_covars, "hypercholesterolemia"),

binary_stats(aurora_patients_covars, "DM2"),

binary_stats(aurora_patients_covars, "smoking"),

binary_stats(aurora_patients_covars, "ICH"),

format_median_iqr(aurora_patients_covars$total)),

sCAA = c(format_age(focas_patients_covars),

binary_stats(focas_patients_covars, "sex"),

binary_stats(focas_patients_covars, "hypertensie"),

binary_stats(focas_patients_covars, "hypercholesterolemia"),

binary_stats(focas_patients_covars, "DM2"),

binary_stats(focas_patients_covars, "smoking"),

binary_stats(focas_patients_covars, "ICH"),

format_median_iqr(focas_patients_covars$total))

)

\# Generate HTML table

html_table <- kable(table1, format = "html",

caption = "Table 1: Patient Characteristics",

col.names = c("Characteristic",

paste0("All Patients (n=", nrow(all_patients_covars), ")"),

paste0("DCAA (n=", nrow(aurora_patients_covars), ")"),

paste0("sCAA (n=", nrow(focas_patients_covars), ")")),

align = "lrrr")

\# Create output directory and save table

if (!dir.exists("output")) {

dir.create("output")

}

output_file <- paste0("output/table1_patient_characteristics_", format(Sys.time(), "%Y%m%d_%H%M%S"), ".html")

cat(as.character(html_table), file = output_file)

print(paste("Table saved as HTML in", output_file))

print("Open this file in a browser, select all, and copy/paste into Word")

\# # -----------------------------------------------------------------

\# # Additional characteristics for JMRI revision June 2026

\# # -----------------------------------------------------------------

summary(covars_no_ICH$sex)

summary(covars_no_ICH$age)

sd(covars_no_ICH$age)

\# # -----------------------------------------------------------------

\# # Table 1 Alternate Version 1: 6-column table (with and without ICH)

\# # -----------------------------------------------------------------

\# Create filtered datasets for no ICH versions

all_patients_covars_no_ICH <- covars %>% filter(!(patient %in% patients_to_exclude_ich))

aurora_patients_covars_no_ICH <- covars %>% filter(mutstat == "aurora" & !(patient %in% patients_to_exclude_ich))

focas_patients_covars_no_ICH <- covars %>% filter(mutstat == "focas" & !(patient %in% patients_to_exclude_ich))

\# # Create 6-column data frame for table

\# table1_6col <- data.frame(

\#	Characteristic = c("Age, years", "Female sex", "Hypertension",

\#	"Hypercholesterolemia", "Diabetes mellitus type 2",

\#	"Smoking, ever", "History of ICH",

\#	"Total CMB, median (IQR)"),

\#	All = c(format_age(all_patients_covars),

\#	binary_stats(all_patients_covars, "sex"),

\#	binary_stats(all_patients_covars, "hypertensie"),

\#	binary_stats(all_patients_covars, "hypercholesterolemia"),

\#	binary_stats(all_patients_covars, "DM2"),

\#	binary_stats(all_patients_covars, "smoking"),

\#	binary_stats(all_patients_covars, "ICH"),

\#	format_median_iqr(all_patients_covars$total)),

\#	All_no_ICH = c(format_age(all_patients_covars_no_ICH),

\#	binary_stats(all_patients_covars_no_ICH, "sex"),

\#	binary_stats(all_patients_covars_no_ICH, "hypertensie"),

\#	binary_stats(all_patients_covars_no_ICH, "hypercholesterolemia"),

\#	binary_stats(all_patients_covars_no_ICH, "DM2"),

\#	binary_stats(all_patients_covars_no_ICH, "smoking"),

\#	binary_stats(all_patients_covars_no_ICH, "ICH"),

\#	format_median_iqr(all_patients_covars_no_ICH$total)),

\#	DCAA = c(format_age(aurora_patients_covars),

\#	binary_stats(aurora_patients_covars, "sex"),

\#	binary_stats(aurora_patients_covars, "hypertensie"),

\#	binary_stats(aurora_patients_covars, "hypercholesterolemia"),

\#	binary_stats(aurora_patients_covars, "DM2"),

\#	binary_stats(aurora_patients_covars, "smoking"),

\#	binary_stats(aurora_patients_covars, "ICH"),

\#	format_median_iqr(aurora_patients_covars$total)),

\#	DCAA_no_ICH = c(format_age(aurora_patients_covars_no_ICH),

\#	binary_stats(aurora_patients_covars_no_ICH, "sex"),

\#	binary_stats(aurora_patients_covars_no_ICH, "hypertensie"),

\#	binary_stats(aurora_patients_covars_no_ICH, "hypercholesterolemia"),

\#	binary_stats(aurora_patients_covars_no_ICH, "DM2"),

\#	binary_stats(aurora_patients_covars_no_ICH, "smoking"),

\#	binary_stats(aurora_patients_covars_no_ICH, "ICH"),

\#	format_median_iqr(aurora_patients_covars_no_ICH$total)),

\#	sCAA = c(format_age(focas_patients_covars),

\#	binary_stats(focas_patients_covars, "sex"),

\#	binary_stats(focas_patients_covars, "hypertensie"),

\#	binary_stats(focas_patients_covars, "hypercholesterolemia"),

\#	binary_stats(focas_patients_covars, "DM2"),

\#	binary_stats(focas_patients_covars, "smoking"),

\#	binary_stats(focas_patients_covars, "ICH"),

\#	format_median_iqr(focas_patients_covars$total)),

\#	sCAA_no_ICH = c(format_age(focas_patients_covars_no_ICH),

\#	binary_stats(focas_patients_covars_no_ICH, "sex"),

\#	binary_stats(focas_patients_covars_no_ICH, "hypertensie"),

\#	binary_stats(focas_patients_covars_no_ICH, "hypercholesterolemia"),

\#	binary_stats(focas_patients_covars_no_ICH, "DM2"),

\#	binary_stats(focas_patients_covars_no_ICH, "smoking"),

\#	binary_stats(focas_patients_covars_no_ICH, "ICH"),

\#	format_median_iqr(focas_patients_covars_no_ICH$total))

\# )

\# # Generate HTML table for 6-column version

\# html_table_6col <- kable(table1_6col, format = "html",

\#	caption = "Table 1 (6-column): Patient Characteristics with ICH Comparison",

\#	col.names = c("Characteristic",

\#	paste0("All Patients (n=", nrow(all_patients_covars), ")"),

\#	paste0("All Patients - No ICH (n=", nrow(all_patients_covars_no_ICH), ")"),

\#	paste0("DCAA (n=", nrow(aurora_patients_covars), ")"),

\#	paste0("DCAA - No ICH (n=", nrow(aurora_patients_covars_no_ICH), ")"),

\#	paste0("sCAA (n=", nrow(focas_patients_covars), ")"),

\#	paste0("sCAA - No ICH (n=", nrow(focas_patients_covars_no_ICH), ")")),

\#	align = "lrrrrrr")

\# # Save 6-column table

\# output_file_6col <- paste0("output/table1_6col_patient_characteristics_", format(Sys.time(), "%Y%m%d_%H%M%S"), ".html")

\# cat(as.character(html_table_6col), file = output_file_6col)

\# print(paste("6-column Table saved as HTML in", output_file_6col))

\# -----------------------------------------------------------------

\# Table 1 Alternate Version 2: 3-column table (no ICH patients only)

\# -----------------------------------------------------------------

\# Create 3-column data frame for no ICH patients only

table1_no_ICH <- data.frame(

Characteristic = c("Age, years", "Female sex", "Hypertension",

"Hypercholesterolemia", "Diabetes mellitus type 2",

"Smoking, ever", "History of ICH",

"Total CMB, median (IQR)"),

All_no_ICH = c(format_age(all_patients_covars_no_ICH),

binary_stats(all_patients_covars_no_ICH, "sex"),

binary_stats(all_patients_covars_no_ICH, "hypertensie"),

binary_stats(all_patients_covars_no_ICH, "hypercholesterolemia"),

binary_stats(all_patients_covars_no_ICH, "DM2"),

binary_stats(all_patients_covars_no_ICH, "smoking"),

binary_stats(all_patients_covars_no_ICH, "ICH"),

format_median_iqr(all_patients_covars_no_ICH$total)),

DCAA_no_ICH = c(format_age(aurora_patients_covars_no_ICH),

binary_stats(aurora_patients_covars_no_ICH, "sex"),

binary_stats(aurora_patients_covars_no_ICH, "hypertensie"),

binary_stats(aurora_patients_covars_no_ICH, "hypercholesterolemia"),

binary_stats(aurora_patients_covars_no_ICH, "DM2"),

binary_stats(aurora_patients_covars_no_ICH, "smoking"),

binary_stats(aurora_patients_covars_no_ICH, "ICH"),

format_median_iqr(aurora_patients_covars_no_ICH$total)),

sCAA_no_ICH = c(format_age(focas_patients_covars_no_ICH),

binary_stats(focas_patients_covars_no_ICH, "sex"),

binary_stats(focas_patients_covars_no_ICH, "hypertensie"),

binary_stats(focas_patients_covars_no_ICH, "hypercholesterolemia"),

binary_stats(focas_patients_covars_no_ICH, "DM2"),

binary_stats(focas_patients_covars_no_ICH, "smoking"),

binary_stats(focas_patients_covars_no_ICH, "ICH"),

format_median_iqr(focas_patients_covars_no_ICH$total))

)

\# Generate HTML table for no ICH version

html_table_no_ICH <- kable(table1_no_ICH, format = "html",

caption = "Table 1 (No ICH): Patient Characteristics - No ICH Patients Only",

col.names = c("Characteristic",

paste0("All Patients - No ICH (n=", nrow(all_patients_covars_no_ICH), ")"),

paste0("DCAA - No ICH (n=", nrow(aurora_patients_covars_no_ICH), ")"),

paste0("sCAA - No ICH (n=", nrow(focas_patients_covars_no_ICH), ")")),

align = "lrrr")

\# Save no ICH table

output_file_no_ICH <- paste0("output/table1_no_ICH_patient_characteristics_", format(Sys.time(), "%Y%m%d_%H%M%S"), ".html")

cat(as.character(html_table_no_ICH), file = output_file_no_ICH)

print(paste("No ICH Table saved as HTML in", output_file_no_ICH))

print("Open these files in a browser, select all, and copy/paste into Word")

\# -----------------------------------------------------------------

\# Figure 1: boxplots of CMB density in different areas

\# -----------------------------------------------------------------

\# Function to create CMB boxplots

create_cmb_boxplot <- function(data, scale_type = "normal", title_suffix = "", filename_suffix = "") {

\# Prepare data for plotting by extracting base area and side

plot_data <- data %>%

mutate(

base_area = str_remove(area, "Left"),

base_area = str_remove(base_area, "Right"),

side = ifelse(str_detect(area, "Left"), "Left", "Right"),

\# Ensure correct ordering on the x-axis

base_area = factor(base_area, levels = c("ACA", "MCA", "PCA")),

side = factor(side, levels = c("Left", "Right"))

)

\# Create y-axis label based on scale type

y_label <- if(scale_type == "log") {

expression('CMB density log1p scale [count per cm'^3*']')

} else if(scale_type == "sqrt") {

expression('CMB density sqrt scale [count per cm'^3*']')

} else {

expression('CMB density [count per cm'^3*']')

}

\# Create base plot

p <- ggplot(plot_data, aes(x = base_area, y = CMB_density, fill = side)) +

geom_boxplot(outlier.shape = NA) + # Creates the boxplots without outlier points

geom_point(aes(color = side), position = position_jitterdodge(jitter.width = 0.4, dodge.width = 0.75),

alpha = 0.8, size = 1.5) + # Add scatter points with proper grouping

labs(

x = "Cerebral arterial flow territory (per hemisphere)",

y = y_label,

fill = "Side", # Sets the legend title for boxes

color = "Side" # Sets the legend title for points

) +

scale_fill_manual(values = c("Left" = "chocolate1", "Right" = "deepskyblue1")) +

scale_color_manual(values = c("Left" = "chocolate4", "Right" = "deepskyblue4")) + # Darker colors for points

guides(color = "none") + # Hide the color legend since fill legend shows the same info

theme_classic() + # Applies the classic R theme

theme(

axis.text = element_text(size = 14), # Axis tick labels

axis.title = element_text(size = 16), # Axis titles

legend.text = element_text(size = 14), # Legend text

legend.title = element_text(size = 16)	# Legend title

)

\# Add scale transformation if requested

if(scale_type == "log") {

p <- p + scale_y_continuous(trans = 'log1p',

breaks = c(0.05, 0.1, 0.25, 0.5, 1.0, 1.5),

labels = scales::label_number())

} else if(scale_type == "sqrt") {

p <- p + scale_y_continuous(trans = 'sqrt',

breaks = c(0.05, 0.1, 0.25, 0.5, 1.0, 1.5),

labels = scales::label_number())

} else {

p <- p + scale_y_continuous(breaks = c(0.05, 0.1, 0.25, 0.5, 1.0, 1.5),

labels = scales::label_number())

}

\# Print the plot to the R graphics device (e.g., RStudio Plots pane)

print(p)

\# Save the plot automatically

if (!dir.exists("output")) {

dir.create("output")

}

\# Save as PNG

filename_png <- paste0("output/figure1", filename_suffix, "_cmb_density_area_boxplot.png")

ggsave(filename_png, plot = p, width = 8, height = 6, dpi = 300)

print(paste("Figure saved to", filename_png))

\# Save as SVG

filename_svg <- paste0("output/figure1", filename_suffix, "_cmb_density_area_boxplot.svg")

ggsave(filename_svg, plot = p, width = 8, height = 6)

print(paste("Figure saved to", filename_svg))

return(p)

}

\# Generate all 6 boxplot variants using the function

#figure1 <- create_cmb_boxplot(counts_density_all, scale_type = "normal", title_suffix = "", filename_suffix = "")

#figure1_log <- create_cmb_boxplot(counts_density_all, scale_type = "log", title_suffix = " (Log Scale)", filename_suffix = "_log")

#figure1_sqrt <- create_cmb_boxplot(counts_density_all, scale_type = "sqrt", title_suffix = " (Sqrt Scale)", filename_suffix = "_sqrt")

#figure1_noICH <- create_cmb_boxplot(counts_density, scale_type = "normal", title_suffix = " (No ICH)", filename_suffix = "_noICH")

#figure1_log_noICH <- create_cmb_boxplot(counts_density, scale_type = "log", title_suffix = " (No ICH, Log Scale)", filename_suffix = "_log_noICH")

#figure1_sqrt_noICH <- create_cmb_boxplot(counts_density, scale_type = "sqrt", title_suffix = " (No ICH, Sqrt Scale)", filename_suffix = "_sqrt_noICH")

\# -----------------------------------------------------------------

\# Supplemental Figure 1: Patient Selection Flowchart

\# This just gives the numbers of patients in each step of the selection process

\# -----------------------------------------------------------------

\# Create data for patient selection flowchart (Updated to reflect actual pipeline)

flowchart_data <- list()

\# Initial dataset

D_initial <- read_xls("data/Lokatie_bloedingen_AURORA_FOCAS_baseline_flowterritory_covariates.xls")

flowchart_data$initial <- length(unique(D_initial$patient))

\# After removing macro bleeds

D_after_macro <- D_initial %>% filter(type != "macro")

flowchart_data$after_macro_removal <- length(unique(D_after_macro$patient))

\# After updating missing smoking values for PATIENT_ID_5

D_after_smoking_update <- D_after_macro %>%

mutate(smoking = case_when(

grepl("^PATIENT_ID_5", patient) ~ 0,

TRUE ~ smoking # Keep existing values for other patients

))

flowchart_data$after_smoking_update <- length(unique(D_after_smoking_update$patient))

\# After removing missing data

D_after_missing <- D_after_smoking_update %>%

filter(mutstat != 9999) %>%

filter(smoking != 9999) %>%

filter(!is.na(smoking))

flowchart_data$after_missing_removal <- length(unique(D_after_missing$patient))

\# After removing patients with history of CAA-ri

D_after_caari <- D_after_missing %>%

filter(!(patient %in% c("PATIENT_ID_1", "PATIENT_ID_2")))

flowchart_data$after_caari_removal <- length(unique(D_after_caari$patient))

\# After removing patients with erroneous CMB counts

D_after_erroneous <- D_after_caari %>%

filter(!(patient %in% c("PATIENT_ID_3", "PATIENT_ID_4")))

flowchart_data$after_erroneous_removal <- length(unique(D_after_erroneous$patient))

\# After removing patients with zero counts (after cerebellar correction)

\# Use the final corrected counts from the actual analysis

zero_count_patients_corrected <- unique(patient_total$patient[patient_total$total == 0])

flowchart_data$after_zero_removal <- length(unique(covars$patient))

\# Final split between D-CAA and sCAA (using the actual final dataset)

flowchart_data$dcaa_patients <- length(unique(covars$patient[covars$mutstat == "aurora"]))

flowchart_data$scaa_patients <- length(unique(covars$patient[covars$mutstat == "focas"]))

\# --- Additions for No ICH counts ---

\# Filter final dataset to exclude ICH patients (using patients_to_exclude_ich defined earlier)

D_final_no_ICH <- covars %>% filter(!(patient %in% patients_to_exclude_ich))

\# Calculate counts for the no ICH group

flowchart_data$after_ICH_removal <- length(unique(D_final_no_ICH$patient))

flowchart_data$dcaa_noICH_patients <- length(unique(D_final_no_ICH$patient[D_final_no_ICH$mutstat == "aurora"]))

flowchart_data$scaa_noICH_patients <- length(unique(D_final_no_ICH$patient[D_final_no_ICH$mutstat == "focas"]))

\# --- End Additions ---

\# Create flowchart dataframe (Structured for visual flowchart creation)

\# Calculate exclusion counts for better flowchart presentation

excluded_missing <- flowchart_data$after_smoking_update - flowchart_data$after_missing_removal

excluded_caari <- flowchart_data$after_missing_removal - flowchart_data$after_caari_removal

excluded_erroneous <- flowchart_data$after_caari_removal - flowchart_data$after_erroneous_removal

excluded_zero_counts <- flowchart_data$after_erroneous_removal - flowchart_data$after_zero_removal

total_first_round_exclusions <- flowchart_data$initial - flowchart_data$after_zero_removal

\# Calculate ICH exclusions

excluded_ICH_total <- flowchart_data$after_zero_removal - flowchart_data$after_ICH_removal

excluded_ICH_dcaa <- flowchart_data$dcaa_patients - flowchart_data$dcaa_noICH_patients

excluded_ICH_scaa <- flowchart_data$scaa_patients - flowchart_data$scaa_noICH_patients

\# Create structured flowchart data

flowchart_structured <- data.frame(

Level = c("Initial", "First_Exclusions", "First_Exclusions", "First_Exclusions",

"First_Exclusions", "First_Exclusions", "Intermediate", "Intermediate",

"Intermediate", "ICH_Exclusions", "ICH_Exclusions", "ICH_Exclusions",

"Final", "Final", "Final"),

Description = c("Initial FOCAS/AURORA dataset",

"Missing data on covariates",

"History of CAA-ri patients",

"Missing CMB masks (erroneous counts)",

"Zero CMB counts (post-cerebellar correction)",

"TOTAL FIRST ROUND EXCLUSIONS",

"Current study cohort",

" - D-CAA mutation carriers",

" - Sporadic CAA patients",

"History of ICH (D-CAA)",

"History of ICH (sCAA)",

"TOTAL ICH EXCLUSIONS",

"Main analysis cohort (No ICH)",

" - D-CAA mutation carriers (No ICH)",

" - Sporadic CAA patients (No ICH)"),

Count = c(flowchart_data$initial,

excluded_missing,

excluded_caari,

excluded_erroneous,

excluded_zero_counts,

total_first_round_exclusions,

flowchart_data$after_zero_removal,

flowchart_data$dcaa_patients,

flowchart_data$scaa_patients,

excluded_ICH_dcaa,

excluded_ICH_scaa,

excluded_ICH_total,

flowchart_data$after_ICH_removal,

flowchart_data$dcaa_noICH_patients,

flowchart_data$scaa_noICH_patients)

)

\# Create a simplified summary table for flowchart creation

flowchart_summary <- data.frame(

Box = c("Initial Dataset", "First Exclusions", "Intermediate Cohort",

"ICH Exclusions", "Final Analysis Cohort"),

Count = c(paste("n =", flowchart_data$initial),

paste(total_first_round_exclusions, "Excluded"),

paste("n =", flowchart_data$after_zero_removal),

paste(excluded_ICH_total, "Excluded"),

paste("n =", flowchart_data$after_ICH_removal)),

Details = c("Initial FOCAS/AURORA dataset",

paste("Missing data (", excluded_missing, "), CAA-ri (", excluded_caari, "), Erroneous masks (", excluded_erroneous, "), Zero counts (", excluded_zero_counts, ")", sep=""),

paste("D-CAA: ", flowchart_data$dcaa_patients, ", sCAA: ", flowchart_data$scaa_patients, sep=""),

paste("D-CAA: ", excluded_ICH_dcaa, ", sCAA: ", excluded_ICH_scaa, sep=""),

paste("D-CAA: ", flowchart_data$dcaa_noICH_patients, ", sCAA: ", flowchart_data$scaa_noICH_patients, sep=""))

)

\# Use the structured version as the main flowchart dataframe

flowchart_df <- flowchart_structured

\# Export to HTML

library(kableExtra)

\# Create detailed structured table

html_table_detailed <- kable(flowchart_structured, format = "html",

caption = "Patient Selection Flowchart - Detailed Structure") %>%

kable_styling(bootstrap_options = c("striped", "hover", "condensed"), full_width = FALSE)

\# Create summary table (better for flowchart creation)

html_table_summary <- kable(flowchart_summary, format = "html",

caption = "Patient Selection Flowchart - Summary for Visual Creation") %>%

kable_styling(bootstrap_options = c("striped", "hover", "condensed"), full_width = FALSE)

\# Combine both tables

combined_html <- paste(

"<h2>Patient Selection Flowchart Data</h2>",

"<h3>Summary Version (for flowchart creation)</h3>",

as.character(html_table_summary),

"<br><br>",

"<h3>Detailed Version (complete breakdown)</h3>",

as.character(html_table_detailed),

sep = ""

)

\# Save HTML file

output_file_flowchart <- paste0("output/flowchart_data_", format(Sys.time(), "%Y%m%d_%H%M%S"), ".html")

writeLines(combined_html, output_file_flowchart)

print(paste("Flowchart data saved to", output_file_flowchart))

\# Print summary to console for easy copying

print("=== FLOWCHART SUMMARY FOR VISUAL CREATION ===")

print(flowchart_summary)

\# -----------------------------------------------------------------

\# Supplemental Figure 2: Histogram of Test Statistic Null Distribution

\# -----------------------------------------------------------------

\# Function to create test statistic histogram

create_test_statistic_histogram <- function(null_statistics, observed_statistic, p_value,

title, fill_color = "steelblue",

binwidth = NULL, filename_prefix = "supplemental_figure",

save_plot = TRUE, use_scale_x_break = TRUE) {

\# Auto-calculate binwidth if not provided

if (is.null(binwidth)) {

binwidth <- diff(range(null_statistics)) / 30 # Default to ~30 bins

}

\# Create the histogram plot

plot <- ggplot(data.frame(statistic = null_statistics), aes(x = statistic)) +

geom_histogram(fill = fill_color, color = "black", binwidth = binwidth) +

geom_vline(xintercept = observed_statistic, linetype = "dashed", color = "#42acd6", size = 1) +

scale_x_continuous(labels = scales::comma) +

scale_y_continuous(expand = expansion(mult = c(0, .05))) +

labs(

title = title,

x = "Test Statistic",

y = "Frequency"

) +

theme_classic() +

theme(axis.text.x.top = element_blank(),

axis.ticks.x.top = element_blank(),

axis.line.x.top = element_blank(),

axis.text = element_text(size = 12), # Axis tick labels

axis.title = element_text(size = 16), # Axis titles

plot.title = element_text(size = 16)	# Plot title

) +

annotate("text",

x = observed_statistic,

y = Inf,

label = paste0("Observed = ", round(observed_statistic, 2), "\np = ", round(p_value, 3)),

hjust = 1.05,

vjust = 1.2,

size = 4) # Consistent with figure 1 text sizing

\# Add scale break if requested and the observed statistic is far from the distribution

if (use_scale_x_break && observed_statistic > max(null_statistics) * 1.05) {

plot <- plot + scale_x_break(c(max(null_statistics) * 1.05, observed_statistic * 0.975))

}

\# Save the plot if requested

if (save_plot) {

if (!dir.exists("output")) {

dir.create("output")

}

\# Save as PNG

filename_png <- paste0("output/", filename_prefix, "_test_statistic_distribution.png")

ggsave(filename_png, plot = plot, width = 8, height = 6, dpi = 300)

print(paste("Figure saved to", filename_png))

\# Save as SVG

filename_svg <- paste0("output/", filename_prefix, "_test_statistic_distribution.svg")

ggsave(filename_svg, plot = plot, width = 8, height = 6)

print(paste("Figure saved to", filename_svg))

}

return(plot)

}

\# -----------------------------------------------------------------

\# Combined Supplemental Figure 2: Null Distributions (No ICH vs All)

\# -----------------------------------------------------------------

\# Create individual histogram plots for combining (without saving individual files)

supplemental_figure_noICH <- create_test_statistic_histogram(

null_statistics = TTs_no_ICH,

observed_statistic = TT_no_ICH,

p_value = p_value_no_ICH,

title = "Distribution of test statistics under null hypothesis (no sICH )",

fill_color = "#5cc836",

binwidth = 200,

filename_prefix = "supplemental_figure_noICH",

save_plot = FALSE, # Don't save individual plots

use_scale_x_break = FALSE

)

supplemental_figure_all <- create_test_statistic_histogram(

null_statistics = TTs,

observed_statistic = TT,

p_value = p_value,

title = "Distribution of test statistics under null hypothesis (all participants)",

fill_color = "#5cc836",

binwidth = 500,

filename_prefix = "supplemental_figure_all",

save_plot = FALSE, # Don't save individual plots

use_scale_x_break = TRUE

)

\# Create the combined figure directly (no title, noICH first)

combined_supplemental_figure <- wrap_plots(supplemental_figure_noICH, supplemental_figure_all, ncol = 1) +

plot_annotation(tag_levels = 'A')

\# Save the combined plot

if (!dir.exists("output")) {

dir.create("output")

}

\# Save as PNG

ggsave("output/supplemental_figure_combined_distributions.png",

plot = combined_supplemental_figure, width = 8, height = 10, dpi = 300)

print("Combined figure saved to output/supplemental_figure_combined_distributions.png")

\# Save as SVG

ggsave("output/supplemental_figure_combined_distributions.svg",

plot = combined_supplemental_figure, width = 8, height = 10)

print("Combined figure saved to output/supplemental_figure_combined_distributions.svg")

print("--- Finished Analysis for No ICH Subset ---")

print("")

print("=================================================================")

print("NULL DISTRIBUTION SYSTEM USAGE:")

print("=================================================================")

print("• To REUSE existing distributions: Leave REGENERATE_NULL_DISTRIBUTIONS commented out")

print("• To GENERATE new distributions: Uncomment 'REGENERATE_NULL_DISTRIBUTIONS <- TRUE'")

print("• Distributions are saved in: data/null_distributions/")

print("• Metadata is tracked in: data/null_distributions/distribution_metadata.csv")

print("• The system automatically validates data consistency")

print("=================================================================")

\# -----------------------------------------------------------------

\# -----------------------------------------------------------------

\# End of main analysis

\# -----------------------------------------------------------------

\# -----------------------------------------------------------------

\# -----------------------------------------------------------------

\# Extra code for informative figure to determine quartiles

\# -----------------------------------------------------------------

\# this code serves to determine quartiles for patients so we know which patients to use for the example figure

\# e.g. a patient in first quartile, median and third quartile

#counts_DCAA <- subset(counts, counts$mutstat == "aurora")

#counts_sCAA <- subset(counts, counts$mutstat == "focas")

#patient_total_DCAA <- counts_DCAA %>% group_by(patient) %>% summarize(total=sum(n))

#patient_total_sCAA <- counts_sCAA %>% group_by(patient) %>% summarize(total=sum(n))

#shapiro.test(patient_total_DCAA$total)

#summary(patient_total_DCAA$total)

#boxplot(patient_total_DCAA$total)

#shapiro.test(patient_total_sCAA$total)

#summary(patient_total_sCAA$total)

#boxplot(patient_total_sCAA$total)

\# -----------------------------------------------------------------

\# ADDITIONAL CODE FOR INFORMING STATISTICIAN ON POWER IN STUDY

\# Create and export covars with ICH and CMB totals

\# Note that this was done to inform statistician on power in the study and is not used in the analysis

\# -----------------------------------------------------------------

\# Select relevant columns from covars (patient, ICH, total)

#covars_subset <- covars %>% select(patient, ICH, total)

\# Join the subset with the pattern dataframe

#covars_ICH_CMB <- pattern %>%

\# left_join(covars_subset, by = "patient")

\# Check if output directory exists, create if not

#if (!dir.exists("output")) {

\# dir.create("output")

#}

\# Export the combined dataframe to CSV

#output_file_covars_csv <- paste0("output/covars_with_ICH_CMB_totals_", format(Sys.time(), "%Y%m%d_%H%M%S"), ".csv")

#write.csv(covars_ICH_CMB, output_file_covars_csv, row.names = FALSE)

#print(paste("Covariate data including ICH status and CMB totals saved to", output_file_covars_csv))

\# Export the combined dataframe to SAV (SPSS)

#output_file_covars_sav <- paste0("output/covars_with_ICH_CMB_totals_", format(Sys.time(), "%Y%m%d_%H%M%S"), ".sav")

#write_sav(covars_ICH_CMB, output_file_covars_sav)

#print(paste("Covariate data including ICH status and CMB totals saved to", output_file_covars_sav))

\# -----------------------------------------------------------------

\# Count occurrences of each covariate combination by microbleeds

\# Note that this was done to inform statistician on power in the study and is not used in the analysis

\# NOTE THAT THIS CURRENTLY DOES NOT INCLUDE THE CEREBELLAR CORRECTION

\# -----------------------------------------------------------------

#---All Patients, including smoking as covar---

\# Count how many rows (bleeds) exist for each covariate combination

#combination_counts_in_expandedcounts <- expandedcounts %>%

\# group_by(combination) %>%

\# summarise(

\#	num_bleeds = n(),

\#	num_patients = n_distinct(patient)

\# ) %>%

\# ungroup() %>%

\# arrange(desc(num_bleeds))

\# Print the counts

#print("Number of bleeds per covariate combination in expandedcounts:")

#print(combination_counts_in_expandedcounts)

\# Optional: Calculate percentage of total bleeds for each combination

#total_bleeds <- sum(combination_counts_in_expandedcounts$num_bleeds)

#combination_counts_in_expandedcounts <- combination_counts_in_expandedcounts %>%

\# mutate(

\#	percentage = round(num_bleeds / total_bleeds * 100, 2),

\#	bleeds_per_patient = round(num_bleeds / num_patients, 2)

\# )

#print("Percentage of total bleeds and bleeds per patient for each combination:")

#print(combination_counts_in_expandedcounts)

\# --- Save Table A1: All Patients, Incl. Smoking ---

#library(kableExtra)

#if (!dir.exists("output")) { dir.create("output") } # Ensure output dir exists

#table_a1_html <- kable(combination_counts_in_expandedcounts, format = "html",

\#	caption = "Table A1: Bleeds per Covariate Combination (All Patients, Including Smoking)",

\#	col.names = c("Covariate Combination", "Number of Bleeds", "Number of Patients", "Percentage (%)", "Bleeds per Patient"),

\#	align = "lrrrr", digits = 2) %>%

\# kable_styling(bootstrap_options = c("striped", "hover", "condensed"), full_width = FALSE)

#output_file_a1 <- paste0("output/table_a1_bleeds_per_combo_all_incl_smoking_", format(Sys.time(), "%Y%m%d_%H%M%S"), ".html")

#cat(as.character(table_a1_html), file = output_file_a1)

#print(paste("Table A1 saved as HTML in", output_file_a1))

#---All Patients, not including smoking as covar---

#combination_counts_no_smoking <- expandedcounts %>%

\# left_join(covars, by = "patient") %>%

\# mutate(combination_no_smoking = paste(mutstat, hypertensie, sex, age10, sep = ":")) %>%

\# group_by(combination_no_smoking) %>%

\# summarise(

\#	num_bleeds = n(),

\#	num_patients = n_distinct(patient)

\# ) %>%

\# ungroup() %>%

\# arrange(desc(num_bleeds))

\# Calculate percentage and bleeds per patient

#total_bleeds_no_smoking <- sum(combination_counts_no_smoking$num_bleeds)

#combination_counts_no_smoking <- combination_counts_no_smoking %>%

\# mutate(

\#	percentage = round(num_bleeds / total_bleeds_no_smoking * 100, 2),

\#	bleeds_per_patient = round(num_bleeds / num_patients, 2)

\# )

#print("Number of bleeds per covariate combination (without smoking):")

#print(combination_counts_no_smoking)

\# --- Save Table A2: All Patients, Excl. Smoking ---

#library(kableExtra)

#if (!dir.exists("output")) { dir.create("output") } # Ensure output dir exists

#table_a2_html <- kable(combination_counts_no_smoking, format = "html",

\#	caption = "Table A2: Bleeds per Covariate Combination (All Patients, Excluding Smoking)",

\#	col.names = c("Covariate Combination", "Number of Bleeds", "Number of Patients", "Percentage (%)", "Bleeds per Patient"),

\#	align = "lrrrr", digits = 2) %>%

\# kable_styling(bootstrap_options = c("striped", "hover", "condensed"), full_width = FALSE)

#output_file_a2 <- paste0("output/table_a2_bleeds_per_combo_all_excl_smoking_", format(Sys.time(), "%Y%m%d_%H%M%S"), ".html")

#cat(as.character(table_a2_html), file = output_file_a2)

#print(paste("Table A2 saved as HTML in", output_file_a2))

#---Only patients without ICH, including smoking as covar---

#combination_counts_no_ICH <- expandedcounts_no_ICH %>%

\# group_by(combination) %>%

\# summarise(

\#	num_bleeds = n(),

\#	num_patients = n_distinct(patient)

\# ) %>%

\# ungroup() %>%

\# arrange(desc(num_bleeds))

\# Calculate percentage and bleeds per patient for the subset

#total_bleeds_subset_no_ICH <- sum(combination_counts_no_ICH$num_bleeds)

#combination_counts_no_ICH <- combination_counts_no_ICH %>%

\# mutate(

\#	percentage = round(num_bleeds / total_bleeds_subset_no_ICH * 100, 2),

\#	bleeds_per_patient = round(num_bleeds / num_patients, 2)

\# )

#print("Number of bleeds per covariate combination (No ICH group, with smoking):")

#print(combination_counts_no_ICH)

\# --- Save Table A3: No ICH, Incl. Smoking ---

#library(kableExtra)

#if (!dir.exists("output")) { dir.create("output") } # Ensure output dir exists

#table_a3_html <- kable(combination_counts_no_ICH, format = "html",

\#	caption = "Table A3: Bleeds per Covariate Combination (No ICH Patients, Including Smoking)",

\#	col.names = c("Covariate Combination", "Number of Bleeds", "Number of Patients", "Percentage (%)", "Bleeds per Patient"),

\#	align = "lrrrr", digits = 2) %>%

\# kable_styling(bootstrap_options = c("striped", "hover", "condensed"), full_width = FALSE)

#output_file_a3 <- paste0("output/table_a3_bleeds_per_combo_noICH_incl_smoking_", format(Sys.time(), "%Y%m%d_%H%M%S"), ".html")

#cat(as.character(table_a3_html), file = output_file_a3)

#print(paste("Table A3 saved as HTML in", output_file_a3))

#---Only patients without ICH,not including smoking as covar---

#combination_counts_no_ICH_no_smoking <- expandedcounts_no_ICH %>%

\# left_join(covars, by = "patient") %>%

\# mutate(combination_no_smoking = paste(mutstat, hypertensie, sex, age10, sep = ":")) %>%

\# group_by(combination_no_smoking) %>%

\# summarise(

\#	num_bleeds = n(),

\#	num_patients = n_distinct(patient)

\# ) %>%

\# ungroup() %>%

\# arrange(desc(num_bleeds))

\# Calculate percentage and bleeds per patient for the subset

#combination_counts_no_ICH_no_smoking <- combination_counts_no_ICH_no_smoking %>%

\# mutate(

\#	percentage = round(num_bleeds / total_bleeds_subset_no_ICH * 100, 2),

\#	bleeds_per_patient = round(num_bleeds / num_patients, 2)

\# )

#print("Number of bleeds per covariate combination (No ICH group, without smoking):")

#print(combination_counts_no_ICH_no_smoking)

\# --- Save Table A4: No ICH, Excl. Smoking ---

#library(kableExtra)

#if (!dir.exists("output")) { dir.create("output") } # Ensure output dir exists

#table_a4_html <- kable(combination_counts_no_ICH_no_smoking, format = "html",

\#	caption = "Table A4: Bleeds per Covariate Combination (No ICH Patients, Excluding Smoking)",

\#	col.names = c("Covariate Combination", "Number of Bleeds", "Number of Patients", "Percentage (%)", "Bleeds per Patient"),

\#	align = "lrrrr", digits = 2) %>%

\# kable_styling(bootstrap_options = c("striped", "hover", "condensed"), full_width = FALSE)

#output_file_a4 <- paste0("output/table_a4_bleeds_per_combo_noICH_excl_smoking_", format(Sys.time(), "%Y%m%d_%H%M%S"), ".html")

#cat(as.character(table_a4_html), file = output_file_a4)

#print(paste("Table A4 saved as HTML in", output_file_a4))

\# -----------------------------------------------------------------

\# -----------------------------------------------------------------

\# End of script

\# -----------------------------------------------------------------

\# -----------------------------------------------------------------
